# Neonatal gut microbiota stratification and identification of SCFA-associated microbial subgroups using unsupervised clustering and machine learning classification

**DOI:** 10.3389/fmicb.2025.1668451

**Published:** 2025-12-04

**Authors:** Payam Hosseinzadeh Kasani, Cheol-Heui Yun, Kee Hyun Cho, Su Jin Jeong

**Affiliations:** 1 Department of Pediatrics, Kangwon National University Hospital, Kangwon National University School of Medicine, Chuncheon, Republic of Korea; 2 Department of Agricultural Biotechnology, and Research Institute of Agriculture and Life Sciences, Seoul National University, Seoul, Republic of Korea; 3 Center for Food and Bioconvergence, and Interdisciplinary Programs in Agricultural Genomics, Seoul National University, Seoul, Republic of Korea; 4 Institutes of Green Bio Science and Technology, Seoul National University, Pyeongchang, Republic of Korea; 5 Department of Pediatrics, CHA Bundang Medical Center, CHA University School of Medicine, Seongnam, Republic of Korea

**Keywords:** neonatal microbiota, microbial clustering, short-chain fatty acids, machine learning classification, unsupervised learning

## Abstract

**Background:**

The neonatal gut microbiome plays a critical role in infant health through the production of short-chain fatty acids (SCFAs). However, the organization of SCFAs-producing microbial communities in neonates remains poorly characterized. This study applied unsupervised clustering and machine learning to classify microbial subgroups associated with SCFAs production, providing insight into their composition and metabolic potential.

**Methods:**

This study recruited 71 mother-infant pairs from Kangwon National University Hospital and Bundang CHA Hospital, collecting meconium samples within five days postpartum. Microbial diversity was analyzed by 16S rRNA gene sequencing (V3–V4 region) at the genus level, and SCFAs were quantified from the same samples. To identify functionally distinct microbial subgroups, K-Means, Agglomerative, Spectral, and Gaussian Mixture Model clustering were applied. Clustering validity was assessed using Silhouette Score, Calinski-Harabasz Index, Davies-Bouldin Index, and Prediction Strength Validation, with t-distributed Stochastic Neighbor Embedding (t-SNE) visualization to evaluate cluster separation. SCFAs distributions across clusters were compared, while random forest and logistic regression models were used to classify SCFAs-associated microbial clusters through Receiver Operating Characteristic curves (ROC).

**Results:**

The clustering analysis identified distinct microbial subgroups linked to SCFAs production, with Agglomerative clustering outperforming K-Means in capturing functionally relevant structures. Cluster 1 had higher SCFAs levels, enriched in *Bacteroides*, *Prevotella*, and *Enterococcus*, while Cluster 2 exhibited lower SCFAs concentrations with a more heterogeneous composition. The introduction of a third cluster in multi-class analysis revealed an intermediate metabolic profile, suggesting a continuum in microbial metabolic function. Classification analysis confirmed random forest model superiority, achieving ROC score of 91.05% (Agglomerative) and 87.74% (K-Means) in binary classification, and 92.98% (Agglomerative) and 89.84% (K-Means) in multi-class classification, demonstrating RF’s strong predictive ability for SCFAs-based clusters.

**Conclusion:**

Unsupervised clustering combined with classification analysis effectively predict SCFAs-associated subgroups and paving the way for future research on longitudinal tracking and functional genomic integration in early-life metabolic health.

## Introduction

The gut microbiome plays a central role in human health, metabolism, and immune regulation, with its composition influenced by environmental factors such as diet and lifestyle (Gacesa et al., 2022; Ahn and Hayes, 2021; Maher et al., 2023). Research indicates that infant gut colonization begins in utero, as bacteria have been detected in the placenta (Guzzardi et al., 2019; Collado et al., 2016), umbilical cord (Jiménez et al., 2005), and meconium of both vaginal and cesarean section-delivered newborns (Dominguez-Bello et al., 2010; Mshvildadze et al., 2010).

A key function of the gut microbiota in human nutrition is the breakdown of complex polysaccharides into simpler sugars, which are then fermented to produce microbial metabolites. Among various microbial metabolites, short-chain fatty acids (SCFAs), particularly butyrate, acetate, and propionate play a crucial role in modulating host metabolism by interacting with G-protein-coupled receptors, which regulate energy balance and immune responses (Koh et al., 2016). They contribute to glucose homeostasis and appetite regulation, directly impacting the risk of obesity and metabolic syndrome (Byrne et al., 2015; Portincasa et al., 2022; Luo et al., 2022). Additionally, SCFAs serve as energy sources for colonocytes, enhancing intestinal epithelial function, strengthening tight junction proteins, and reducing intestinal permeability, which is particularly important in preventing conditions like necrotizing enterocolitis (Gao et al., 2021; den Besten et al., 2013). Their anti-inflammatory properties further support gut health, making them essential in dietary interventions for inflammatory bowel disease (Parada Venegas et al., 2019; Liu et al., 2021). Emerging research also suggests that SCFAs regulate epigenetic modifications through histone deacetylase inhibition, linking gut microbiota activity to gene expression changes associated with metabolic diseases (Kopczyńska and Kowalczyk, 2024; Nshanian et al., 2025; Guo et al., 2022).

The significance of SCFAs extends to early life, where play a crucial role in infant health by shaping gut microbiota, supporting immune development, and influencing metabolic pathways, as they help maintain gut homeostasis by fueling colonic cells, strengthening the gut barrier, and regulating inflammation (Bridgman et al., 2017; Heath et al., 2020; Barman et al., 2024; Milani et al., 2017). Additionally, emerging research suggests SCFAs contribute to neuroprotection and cognitive development (Hernández-Martínez et al., 2022; Łoniewska et al., 2023).

Accumulating evidence highlights the potential health benefit of SCFAs in pediatric populations and disruptions in gut microbiota composition and SCFAs production are increasingly associated with a range of pediatric health issues, including obesity, allergic disorders, inflammatory bowel disease, and neurodevelopmental disorders (Hernández-Martínez et al., 2022; Abo Kouadio Jerome et al., 2023; Cifuentes et al., 2024; Chun and Toldi, 2022; Hsu et al., 2024; Gio-Batta et al., 2022).

This is especially important because they primarily rely on breast milk components such as human milk oligosaccharides, lactose, and lipids, with SCFAs providing an additional energy source, particularly in preterm or low-birth-weight infants (Grier et al., 2017; Dai et al., 2020; Mueller et al., 2021). The detection of SCFAs within the first a weeks of life indicates that microbial colonization occurs rapidly, with bacteria ferment available substrates to produce metabolic byproducts (Milani et al., 2017; Mueller et al., 2021; Wang et al., 2021). This aligns with studies showing that *Bifidobacterium*, *Bacteroides*, and *Lactobacillus* establish early metabolic activity, reinforcing their role functionally active organisms rather than transient colonizers (Tamburini et al., 2016).

Given the high interindividual variability in gut microbial composition, classifying microbial communities based on functional metabolic outputs rather than taxonomy alone is crucial for understanding early-life microbiome development (Luo et al., 2025; Favari et al., 2024). Neonatal gut communities are highly dynamic and transitional, making it challenging to identify functionally relevant microbial subgroups using traditional taxonomic methods alone. Clustering analysis offers a powerful unsupervised machine learning approach to uncover patterns in microbial composition that may correlate metabolic activities (Reitmeier et al., 2020; Cai et al., 2017; Cai and Sun, 2011). Traditional microbiome studies often focus on relative bacterial abundances, overlooking functional redundancy and microbial interactions, which are essential for understanding gut microbial metabolism and host-microbiome interactions (Turnbaugh et al., 2009; Wu et al., 2020). By applying clustering algorithms to microbial dataset, researchers can identify novel microbiome subtypes linked to metabolic outputs, offering deeper insights into microbial functionality beyond taxonomic classification. Despite advances in microbiome-based clustering, several key knowledge gaps remain:

- SCFAs are widely studied in adults, but their microbiome relationships in neonates remain unclear, particularly in the early postnatal period when microbial colonization is evolving rapidly.
- The extent to which microbial clustering reflects functional metabolic differences is unknown, as the neonatal gut microbiome is still developing, making it difficult to distinguish stable metabolic signatures from transient colonization patterns (Bäckhed et al., 2015).
- While clustering methods have been applied to adult gut microbiome studies, their use in neonatal microbiomes remains scarce, limiting our understanding of whether microbial structures in neonates align with functional SCFAs production.

Addressing these gaps could provide new insights into early-life microbiome functionality, and thus we hypothesize that neonatal gut microbiota can be classified into distinct functional subgroups with varying SCFAs production capacities, each exhibiting specific microbial signatures predictive of metabolic function.

This study aims to classify neonatal gut microbiota into functionally relevant subgroups to determine whether distinct microbial clusters exhibit differential SCFAs profiles. Using unsupervised clustering based solely on genus-level microbial composition, without including any maternal or infant metadata, we sought to identify intrinsic microbiome structures independent of external clinical factors. With multiple clustering techniques including K-Means, Agglomerative Clustering, Spectral Clustering, and Gaussian Mixture Models (GMM), we classify microbial communities based on genus-level taxonomic composition and assess their correlation with SCFAs variations. To determine the most effective clustering approach, Silhouette Score, Calinski-Harabasz Index, Davies-Bouldin Index, and Prediction Strength Validation will be used, alongside t-distributed Stochastic Neighbor Embedding (t-SNE) visualization for stability assessment. We identified key microbial genera contributing to high or low SCFAs production and examined whether microbial communities followed a binary or multi-clustered metabolic structure. To evaluate the functional relevance of SCFAs clusters, we applied supervised machine learning models, including Logistic Regression and Random Forest, to determine whether different level of SCFAs could be accurately predicted. By integrating unsupervised clustering with SCFAs profiling, this study identifies functionally distinct microbial subgroups that may influence neonatal metabolism, immunity, and gut health. These findings could enhance understanding of microbial colonization, aid in early diagnosis of microbiome-related disorders, and support targeted interventions to optimize SCFAs production and improve long-term health outcomes.

## Materials and methods

### Study design and participants

This study included a subset of 71 healthy mother-infant pairs recruited from Kangwon National University Hospital and Bundang CHA Hospital to study the establishment of gut microbiota during infancy. Seventy-one participants, recruited between December 06, 2021 and January 11, 2022, were asked to collect fecal samples from their infants. The study was approved by the Institutional Review Board of Kangwon National University Hospital (IRB no. KNUH-B-2021-12-004) for medical research. The approvals, as well as informed consent from the mothers, were obtained prior to collection of data and samples. Only healthy, full-term newborns (gestational age from 37 weeks 0 days to 41 weeks 6 days) with a birth weight of 2,500 grams or more, admitted to the newborn nursery, were included in the study. Infants admitted to the neonatal intensive care unit (NICU) were excluded, with the exception of those admitted for hyperbilirubinemia after 48 h of life, provided that phototherapy was the only treatment required. Mothers with a history of prolonged rupture of membranes (PROM) lasting 18 h or more, preterm labor, or antibiotics use during pregnancy were excluded to control for variables that could influence maternal and neonatal microbiome composition and health outcomes. Among the 71 enrolled infants, 43 were female and 28 were male. The majority were delivered by Cesarean section (*n* = 68), while only three infants were born via vaginal delivery. Twin births accounted for five cases of the cohort. Maternal conditions assessed included gestational diabetes mellitus (GDM; *n* = 7) and pregnancy-induced hypertension (PIH; *n* = 3). Baseline characteristics of the enrolled infants and their mothers are summarized in Table 1.

**Table 1:** Baseline characteristics of enrolled infants and mothers.

Variable	*n* (%) or Mean ± SD	Median [IQR]	(Min–Max)
Sex (Female)	43 (60.6%)	—	—
Sex (Male)	28 (39.4%)	—	—
Delivery mode (Vaginal)	3 (4.2%)	—	—
Delivery mode (Cesarean)	68 (95.8%)	—	—
Gestational age (weeks)	38.51 ± 0.84	38.60 [38.0–39.0]	36.90–40.6
Birth weight (g)	3172.61 ± 302.82	3,170 [2980–3,385]	2,500–4,130
Maternal age (years)	34.13 ± 4.4	34.00 [31.50–37.50]	24.0–26.0
Maternal BMI at delivery	28.00 ± 3.96	27,051 [25.40–30.04]	20.16–39.82
GDM (Yes)	7 (9.9%)	—	—
PIH (Yes)	3 (4.2%)	—	—
Twin (Yes)	5 (7%)	—	—

Baseline characteristics of the study population (*n* = 71). Continuous variables are presented as mean ± SD, median [IQR], and range; categorical variables as counts and percentages. SCFA concentrations are expressed in μmol/g of stool.

n, number of infants included in the analysis. No missing data were observed for any variable. BMI, body mass index; GDM, gestational diabetes mellitus; PIH, pregnancy-induced hypertension.

### Sample collection and storage

For fecal sample collection, meconium samples were collected from 71 infants within 5 days after birth using a fecal sampling kit of spoon type (Noble Bio®). The samples were immediately stored at −20 °C by hospital staff and then transported to the laboratory, where they were stored at −80 °C until analysis.

### DNA extraction and metabarcoding

DNA was extracted using TIANLONG®-nucleic acid extraction kit (for stool DNA/RNA extraction) then determined concentration and purity using DNA/protein Analyzer (Pultton®). The target region (V3-V4) was amplified using PCRBIO VeriFi Mix (PCR Biosystems®) and 16S-amplicon primer (Macrogen®) at 95 °C for 3 min hot start followed by 25 cycles of 95 °C for 30s and 55 °C for 30s, 72 °C for 30s with a final elongation step of 72 °C for 5 min. The amplified DNA subsequently were purified automatically in nucleic acid extractor (TINLONG® Libex) using MagListo™ PCR/Gel Purification Kit (Bioneer®). Afterwards, Index PCR was performed to attach in dual indices and Illumina sequencing using PCRBIO VeriFi Mix (PCR Biosystems®) and Nextera® Index kit V2 Set A and Set B (Illumina®) at 95 °C for 3 min hotstart followed by 8 cycles of 95 °C for 30s and 55 °C for 30s, 72 °C for 30s with a final elongation step of 72 °C for 5 min. After indexing, the libraries were cleaned up using the same method and the size and concentration were determined using Qsep100 (Bioptic®) and Qbit flex fluorometer (Invitrogen®). After Qbit and Qsep measurements were completed, the mixing volume value was calculated then the PCR product was pooled into a microtube. The pooled libraries were denatured with NaOH 0.2 N, and then diluted with hybridization buffer before sequencing. Finally, DNA were pooled and sequenced using MiSeq® Reagent Kit V3 600 cycles kit (Illumina®) on an Illumina Miseq platform according to the manufacturer’s standard instruction. PhiX was used as an internal control. The V3-V4 hypervariable regions of the bacterial 16S rRNA gene were amplified using the following primers: forward primer 341F (5’-CCTACGGGNGGCWGCAG-3′) and reverse primer 805R (5’-GACTACHVGGGTATCTAATCC-3′) (PMID: 22933715). Sequencing data were analyzed using the DADA2 pipeline (version 1.16) (PMID: 27214047). Briefly, read trimming and filtering were performed using the filterAndTrim function in DADA2. Specifically, reads were truncated at any site with a quality score below 2 and reads with more than two expected errors were discarded. Additionally, reads were trimmed to remove primers and low-quality tails, with forward reads trimmed to 240 bp and reverse reads to 160 bp, based on the quality profiles of our sequencing runs. After trimming, the forward and reverse reads retained an overlap of approximately 25 bp, which was sufficient for accurate merging using the mergePairs() function in DADA2. The merged reads reconstructed full-length amplicons of approximately 430–440 bp, consistent with the expected length of the V3–V4 region. Amplicon Sequence Variants (ASVs) were inferred directly from these merged reads following DADA2’s standard denoising pipeline, and no artificial concatenation of non-overlapping ends was performed. Reads shorter than 150 base pairs were discarded. DADA2’s error model was then used to learn the error rates from the data, which were subsequently used to denoise the sequences. Forward and reverse reads were merged to create full-length sequences of the V3-V4 region. Chimeric sequences were identified and removed using the remove BimeraDenovo function in DADA2. An Amplicon Sequence Variant (ASV) table including 192,815 sequences was created, representing the abundance of each unique sequence in each sample. ASVs were assigned taxonomy using the SILVA database (release 138) (PMID: 23193283) with the assign Taxonomy function in DADA2. Taxonomic assignment results showed high resolution across different taxonomic levels. At the Kingdom level, 99.4% of ASVs were successfully assigned, all of which belonged to the Bacteria kingdom. Additionally, over 99% of ASVs were assigned at the Phylum, Class, Order, and Family levels.

### Short-chain fatty acids analysis

Fecal samples were collected directly from infant diapers using sterile swabs, transferred to cryogenic tubes, and immediately stored at −20 °C until transport to the laboratory. For SCFA quantification, approximately 50 mg of fecal material was suspended in 800 μL of deionized water and acidified with 10 μL of 5 M HCl. The mixture was vortexed, followed by extraction with 400 μL of diethyl ether under cold conditions for 5 min. After centrifugation at 14000 rpm for 1 min, 200 μL of the ether layer was transferred to a clean tube, mixed with 20 μL of N, O-Bis(trimethylsilyl)trifluoroacetamide (BSTFA), and derivatized at 70 °C for 20 min, then incubated at 37 °C for 2 h. The resulting derivatives were analyzed by gas chromatography–mass spectrometry (GC–MS).

SCFAs were quantified using a Shimadzu GC-2010 Plus system coupled to a GCMS-TQ8030 detector (Tokyo, Japan) equipped with a DB-5 ms capillary column (30 m × 0.25 mm i.d., 0.25 μm film; Agilent J&W Scientific, Folsom, CA, USA). One microliter of the derivatized sample was injected in split mode (50:1) at 200 °C, with an oven temperature program as follows: hold at 40 °C for 2 min, ramp to 70 °C at 10 °C/min, to 85 °C at 4 °C/min, to 110 °C at 6 °C/min, and finally to 290 °C at 9 °C/min, maintained for 6 min. Helium served as the carrier gas at a constant flow rate of 0.89 mL/min. The ion source and interface temperatures were set at 200 °C and 250 °C, respectively. Data were collected in scan mode with an electron impact voltage of 0.1 kV and an event time of 0.03 s, monitoring the following m/z ions: 117 (acetic acid), 131 (propionic acid), and 145 (butyric acid). Quantification was performed using calibration curves generated from analytical standards of acetic acid (JUNSEI Chemical Co., Tokyo, Japan), propionic acid (Sigma-Aldrich, St. Louis, MO, USA), and butyric acid (Sigma-Aldrich). This analytical procedure followed the validated protocol previously described in our earlier study (Kwon et al., 2024) and was applied here with identical parameters for methodological consistency.

### Diversity and abundance analysis

Alpha diversity was assessed at the genus level using Chao1 richness and Shannon diversity, which, respectively, estimate species richness and capture both richness and evenness of microbial communities. Prior to diversity analysis, the ASV table was processed in R (v4.0) using the phyloseq (v1.42) (McMurdie and Holmes, 2013) and microbiome (v1.18) (Lahti and Shetty, 2017) packages. Count data were rounded to the nearest integer, and zero-count taxa were removed to avoid distortions caused by rare or uninformative features and no rarefaction was applied. Both Chao1 and Shannon indices were calculated using the estimate_richness() function in phyloseq, and their distributions were compared among clusters using Kruskal–Wallis and Wilcoxon rank-sum tests.

For beta diversity, community dissimilarity among samples was quantified using Euclidean distances computed on Centered Log-Ratio (CLR)–transformed genus-level data, following compositional data analysis principles. The CLR transformation was implemented in R/Bioconductor package (Sharma et al., 2020) to account for the compositional nature of sequencing data. Distance matrices were generated with the *vegan* R package (v2.5–7) (Oksanen et al., 2001) and used to evaluate overall differences in microbial community composition between clusters. Ordination was performed using Principal Coordinates Analysis (PCoA) implemented in the ordinate() function of *phyloseq* (McMurdie and Holmes, 2013), and results were visualized with *ggplot2* (v3.4) (Wickham, 2016). Ellipses representing the 95% confidence interval for each cluster were added to assess separation in community structure. Statistical differences in microbial composition were tested using PERMANOVA (Permutational Multivariate Analysis of Variance) with the adonis2() function in *vegan* (999 permutations). The resulting *p*-values and R^2^ values indicated the significance and proportion of variation explained by the cluster groupings. All plots were generated using consistent color palettes and shape encodings to facilitate visual comparison across clustering methods.

For microbial composition analysis, genus-level abundance tables were quality-filtered, and missing values were replaced with zeros. To normalize for differences in sequencing depth, raw counts were converted into relative abundances (%) by dividing each genus count by the total number of reads per sample and multiplying by 100. The normalized abundance data were reshaped into long format in R using the tidyverse package (Wickham et al., 2019), and stacked bar plots were created using ggplot2 to visualize the distribution of dominant genera across K-Means and Agglomerative clusters. Each bar represents an individual sample, while colors correspond to bacterial genera grouped by phylum. This approach ensured direct comparability of taxonomic composition among clusters while maintaining the proportional structure of microbial communities.

### Machine learning analysis

To investigate the relationship between genus-level microbial abundance clustering and SCFAs concentrations, a combination of supervised and unsupervised machine learning models was applied. Unsupervised clustering was used to identify naturally occurring patterns in microbial composition, grouping infants based on their gut microbiota profiles. This allowed for the exploration of potential associations between microbial clusters and SCFAs levels. Following this, supervised machine learning models were employed to assess the predictive power of microbial composition in distinguishing SCFAs-driven clusters, enabling a deeper understanding of the microbial signatures linked to SCFAs variation. By integrating both approaches, this analysis aimed to uncover biologically meaningful patterns in microbial community structure and their potential influence on metabolic outputs.

### Unsupervised machine learning

Unsupervised clustering analysis was performed exclusively on infant gut microbial relative abundances at the genus level to identify natural groupings within the dataset. No infant or maternal metadata (e.g., delivery mode, feeding type, or gestational age) were included in the clustering process to ensure that the resulting groups reflected intrinsic microbial community structure rather than external influences.

Four clustering algorithms were applied including: K-Means, Agglomerative Hierarchical Clustering, Spectral Clustering, and Gaussian Mixture Model (GMM), each offering distinct advantages in detecting underlying patterns in microbial composition. To determine the optimal number of clusters, multiple evaluation metrics were utilized, including the Silhouette Score, which measures cluster cohesion and separation; the Calinski-Harabasz Index, which evaluates the compactness and separation of clusters; the Davies-Bouldin Index, which quantifies cluster dispersion; and Prediction Strength Validation, which assesses the stability of the clustering results. These complementary metrics ensured the selection of biologically meaningful and well-separated clusters for further downstream analysis. Both binary (two-cluster) and multi-class (three-cluster) configurations were examined based on performance consistency and interpretability. To visualize clustering patterns, the t-distributed Stochastic Neighbor Embedding (t-SNE) algorithm was applied to reduce high-dimensional microbial data into two dimensions, providing an intuitive overview of cluster separation.

After determining the optimal clustering structure based on genus-level microbial composition, a cluster label was assigned to each infant sample. This cluster information was then merged with the corresponding SCFA concentration data and maternal–infant metadata by matching sample identifiers. This post-clustering integration allowed us to compare metabolic (SCFA) and clinical variables across microbiome-derived clusters, while ensuring that only microbial features influenced the initial clustering process. In other words, SCFA and clinical data were used solely for downstream interpretation, not for model training or cluster formation, thereby avoiding bias and preserving the unsupervised nature of the analysis.

### Supervised machine learning

To evaluate the discriminative potential of SCFAs clusters, supervised machine learning models were employed to classify samples based on their microbial composition and SCFAs profiles. For this purpose, two well-established classifiers were utilized, each offering distinct advantages in handling classification tasks as summarized blow.

Logistic regression (LR) is a statistical method adapted for ML that models the probability of an outcome based on input variables, primarily used for classification by applying a sigmoid function to a linear combination of features (Cox, 1958). While LR excels in distinguishing linearly separable categories, it can be extended to multiclass classification. Random forest (RF), a widely used supervised ML algorithm, employs ensemble learning by constructing multiple decision trees to improve classification and regression accuracy (Tin Ho, 1995). Each tree produces a class prediction, and the final outcome is determined by majority voting, making RF highly robust and versatile for complex predictive tasks.

To optimize model performance, Grid Search with Cross-Validation (GridSearchCV) was employed to fine-tune hyperparameters and identify the best-performing configurations (Arlot and Celisse, 2010). The dataset was divided into stratified folds, where one-fold served as the test set while the remaining folds were used for training. A 5-fold cross-validation approach was applied to ensure all data points were evaluated across multiple iterations, reducing bias and improving generalizability. The optimal hyperparameters were selected based on the highest Area Under the Curve (AUC) score in the validation set. Given the potential issue of class imbalance, the class_weight parameter in scikit-learn was applied to adjust for disparities in sample distribution, assigning higher weights to smaller sample classes and lower weights to larger sample classes to prevent bias in predictions (Mueller, n.d.).

Model performance was evaluated using the Receiver Operating Characteristic (ROC) curve, a widely used graphical tool for assessing the performance of binary and multi-class classification models across varying decision thresholds (Tan, 2009). The ROC curve plots the true positive rate (sensitivity) against the false positive rate (1-specificity), providing insight into the trade-off between sensitivity and specificity at different classification thresholds. It is particularly useful for comparing classifiers, as it remains independent of class distribution and enables performance assessment across multiple threshold values.

### Statistical analyses

Sequencing data analysis was conducted using R version 4.0, utilizing the phyloseq and ggplot2 packages (R Core Team, 2020; McMurdie and Holmes, 2013; Wickham, 2016). The top abundances of the microbiome were visualized using the aggregate top taxa and plotting functions provided by the microbiome package (Lahti and Shetty, 2017). The programming tasks in this study were executed using Python programming language (version 3.9) (Van Rossum and Drake, 2009). For data preprocessing and analysis, we utilized Pandas (McKinney, 2010) and NumPy (Harris et al., 2020), two popular Python libraries for data manipulation and analysis. Additionally, Scikit-learn (Pedregosa et al., 2011), a Python module that incorporates various ML algorithms, was employed for the analysis. We performed all analyses on 24-core Intel(R) Xeon(R) Gold 5,118 CPU @ 2.30GHz, RAM 128 GB (Intel Corporation, Santa Clara, CA, United States) running Windows 10 Pro. The data were expressed as means and standard deviations (continuous variables) or as numbers and percentages (categorical variables).

## Results

**Figure 1:**
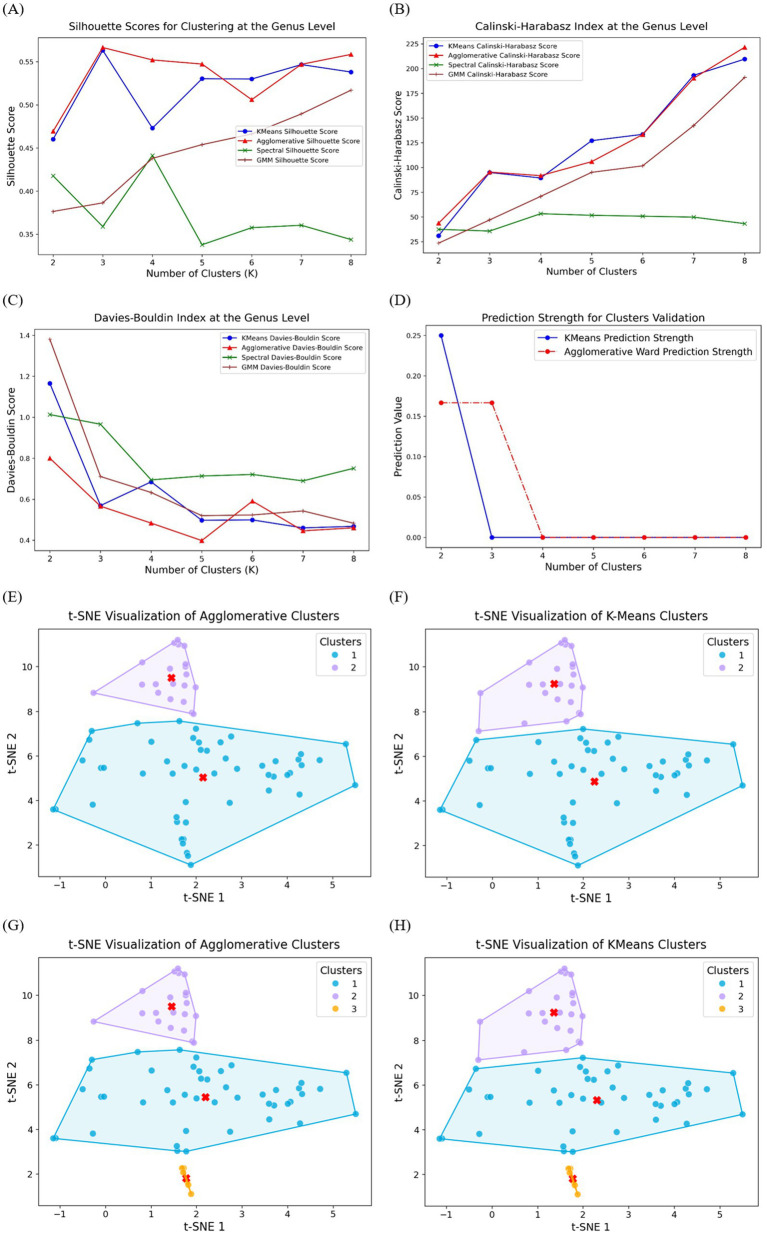
Clustering performance evaluation at the genus level using four clustering models: K-Means, Agglomerative, Spectral, and Gaussian Mixture Model. **(A)** Silhouette scores, where higher values indicate better-defined clusters; **(B)** Calinski–Harabasz index, with higher scores reflecting more compact and well-separated clusters;**(C)** Davies–Bouldin index, where lower values indicate superior clustering performance; **(D)** Prediction strength analysis for K-Means and Agglomerative models. Performance of t-SNE implementation and cluster visualization: **(E)** Agglomerative binary clusters; **(F)** K-Means binary clusters; **(G)** Agglomerative multi-clusters; **(H)** K-Means multi-clusters. The convex hulls encapsulate each cluster, providing a visual representation of their boundaries, and the centroids, marked in red, highlight the central tendency of each cluster.

Figure 1A presents the silhouette scores for clustering at the genus level using four clustering models. The Agglomerative and K-Means models achieved the highest silhouette scores across different cluster numbers, particularly for *K* = 3, where the Agglomerative model outperformed the others. The GMM model demonstrated moderate performance, while the Spectral clustering method consistently exhibited the lowest silhouette scores, suggesting weaker cluster separation.

To further assess clustering quality, the Calinski-Harabasz index was computed, as shown in Figure 1B. Similar to the silhouette scores, the Agglomerative and K-Means models demonstrated superior performance, showing a consistent increase in the Calinski-Harabasz index as the number of clusters increased. Notably, the Spectral clustering method yielded substantially lower scores across all K values, reinforcing its lower clustering efficiency at the genus level. The GMM model displayed intermediate performance, ranking below Agglomerative and K-Means but above Spectral clustering.

The Davies-Bouldin index, an internal validation metric where lower scores indicate better clustering, is presented in Figure 1C. The Agglomerative and K-Means models again achieved the lowest Davies-Bouldin scores across most cluster numbers, with K-Means performing slightly better at *K* = 3. The GMM model exhibited relatively higher scores but still performed better than the Spectral method, which consistently yielded the highest Davies-Bouldin values, reflecting poor cluster compactness and separation.

Since Agglomerative and K-Means models performed better in previous three tests, prediction strength was evaluated for K-Means and Agglomerative clustering, as shown in Figure 1D, to validate cluster stability. Both methods exhibited strong prediction values at *K* = 2 where K-Means achieved higher prediction strength score, but a sharp decline was observed for higher K values, particularly beyond *K* = 3. The Agglomerative method maintained slightly higher prediction strength at *K* = 3 before decreasing, suggesting that three clusters may be a suitable choice for genus-level classification. This aligns with the results from silhouette, Calinski-Harabasz, and Davies-Bouldin scores, reinforcing the robustness of the clustering performance at *K* = 3.

Based on the clustering performance evaluation (Figure 1), Agglomerative and K-Means clustering were identified as the most effective models for grouping the dataset. Given the limited sample size (71 samples), we focused on two-clusters and three-clusters solutions to achieve both broad and fine-grained classifications.

**Figure 2:**
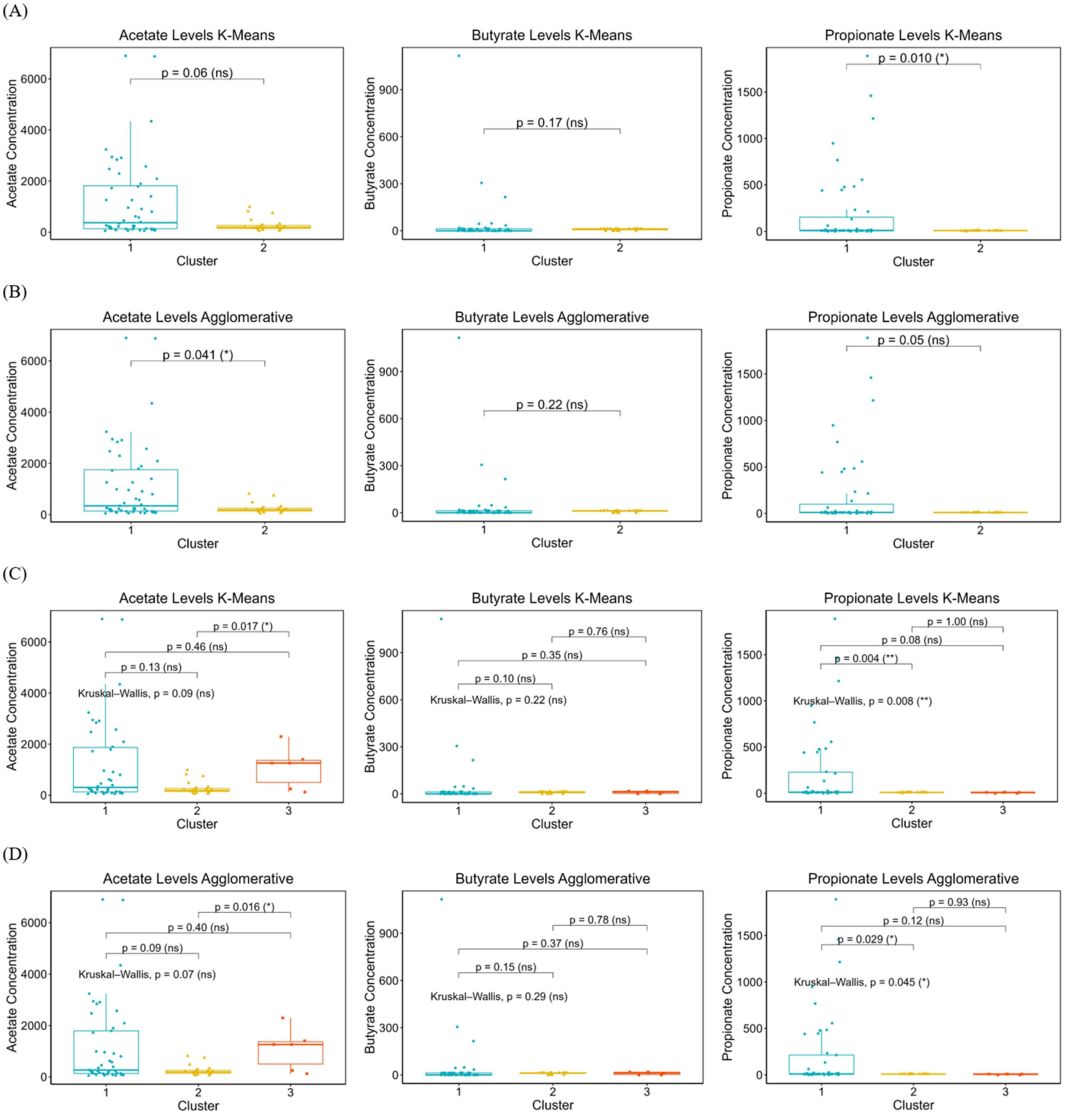
The distribution of SCFAs concentrations (acetate, butyrate, and propionate) across clusters identified by K-Means and Agglomerative clustering methods. **(A)**: the SCFAs levels in binary clustering using K-Means, **(B)** SCFAs levels in binary clustering using Agglomerative, **(C)** SCFAs levels in multi-clustering using K-Means, **(D)** SCFAs levels in multi-clustering using Agglomerative. Kruskal-Wallis tests were used to assess overall differences across clusters, with pairwise comparisons for significant contrasts. Boxplots display median, interquartile range, and data distribution, highlighting potential differences in SCFAs production among microbial subgroups. Statistically significant differences are marked with asterisks (*p < 0.05, *p* < 0.01), while non-significant comparisons are labeled as ns (not significant).

Figures 1E,F illustrates the t-SNE visualization of Agglomerative and K-Means models with two clusters, where the data points are clearly separated into two distinct groups. This clustering approach successfully captured the structure within the dataset, with one group forming a compact cluster at the upper region, while the other cluster was more widely distributed. The clear separation of clusters suggests that Agglomerative model effectively identifies major subgroup differences at this level, aligning with the clustering validation metrics observed earlier. Figures 1G,H present the three-cluster solutions for Agglomerative and K-Means clustering, respectively. The introduction of a third cluster resulted in further subdivision within the data structure. In both clustering methods, the newly identified third cluster is positioned in the lower region of the t-SNE space, suggesting that this group exhibits distinct characteristics from the other two. While Agglomerative clustering (Figure 2C) maintained a hierarchical organization, K-Means clustering (Figure 2D) introduced a more compact third cluster, highlighting slight methodological differences in cluster assignment. These visualizations confirm that the three-cluster approach enhances granularity in data interpretation, further refining subgroup differentiation within the dataset.

To determine whether the identified microbial clusters exhibited distinct SCFAs profiles, SCFAs concentrations were compared across binary and multi-cluster solutions as presented in Table 2. This analysis aimed to assess whether microbial community structure correlates with SCFAs levels, providing insight into potential microbial metabolic functions. In both K-Means and Agglomerative binary clustering, cluster 1 exhibited significantly higher levels of SCFAs compared to cluster 2, which showed markedly lower concentrations. Specifically, in K-Means model, cluster 1 had the highest acetate (1190.57), butyrate (41.08), and propionate (200.86) levels, while cluster 2 showed much lower values (acetate: 280.57, butyrate: 9.36, propionate: 8.90). A similar pattern was observed in Agglomerative model, where cluster 1 demonstrated elevated acetate (1147.60), butyrate (39.17), and propionate (189.39) levels, whereas cluster 2 exhibited substantially lower values (acetate: 253.64, butyrate: 9.48, propionate: 9.34). These results suggest that cluster 1 represents a group with high SCFAs concentrations, whereas cluster 2 corresponds to a low SCFAs profile.

**Table 2:** Comparison of SCFAs levels across clusters identified by K-Means and Agglomerative models.

	K-Means		Agglomerative	
Binary clusters	1 (*n* = 48)	2 (*n* = 23)	1 (*n* = 51)	2 (*n* = 20)
Acetate	1190.57	280.57	1147.60	253.64
Butyrate	41.08	9.36	39.17	9.48
Propionate	200.86	8.90	189.39	9.34

	K-Means			Agglomerative		
Multi clustering	1 (*n* = 42)	2 (*n* = 23)	3 (*n* = 6)	1 (*n* = 45)	2 (*n* = 20)	3 (*n* = 6)
Acetate	1203.75	280.57	1098.30	1154.17	253.64	1098.3
Butyrate	45.50	9.36	10.19	43.03	9.48	10.19
Propionate	228.65	8.90	6.32	213.8	9.34	6.32

The SCFAs concentrations (acetate, butyrate, and propionate) are presented for binary and multi-clustering solutions using K-Means and Agglomerative clustering methods. Clusters were derived based on genus-level microbial composition, and SCFAs levels are reported as mean values within each cluster. Binary clustering represents two distinct microbial groups, whereas multi-clustering further divides the dataset into three clusters.

In K-Means and Agglomerative multi-clustering, subtle variations in SCFAs levels are more apparent. In both methods, cluster 1 consistently exhibited the highest SCFAs levels, with K-Means values of acetate (1203.75), butyrate (45.50), and propionate (228.65), and Agglomerative values of acetate (1154.17), butyrate (43.03), and propionate (213.80). This indicates that cluster 1 represents individuals with elevated SCFAs concentrations. Meanwhile, cluster 2 displayed the lowest SCFAs levels, with acetate, butyrate, and propionate concentrations significantly reduced in both K-Means (280.57, 9.36, 8.90) and Agglomerative clustering (253.64, 9.48, 9.34), suggesting that Cluster 2 represents individuals with a low SCFAs profile. Cluster 3 demonstrated moderate SCFAs levels, positioned between the high and low clusters. In both clustering methods, acetate (K-Means: 1098.30, Agglomerative: 1098.30), butyrate (10.19), and propionate (6.32) were slightly higher than the lowest cluster but did not reach the elevated levels of cluster 1. This suggests that cluster 3 may represent an intermediate SCFAs profile, potentially reflecting a transitional or mixed metabolic state. These findings indicate a clear stratification of SCFAs levels across the three clusters, with cluster 1 being the highest, cluster 2 the lowest, and cluster 3 exhibiting moderate SCFAs concentrations. The corresponding maternal and neonatal characteristics associated with each clustering method are summarized in Supplementary Table 1 (binary clustering) and Supplementary Table 2 (multi-class clustering).

Figures 2A,B, present the SCFAs levels across two clusters identified using K-Means clustering. The propionate levels differ significantly between the two clusters [*p* = 0.010 (*)], while acetate (*p* = 0.06, ns) and butyrate (*p* = 0.17, ns) do not show statistically significant differences.

While in Agglomerative model, the statistical results reveal a significant difference in acetate levels between the two clusters [*p* = 0.041 (*)], whereas propionate (*p* = 0.05, ns) and butyrate (*p* = 0.22, ns) do not show strong evidence of clustering effects. Figure 2C present the boxplot distributions of acetate, butyrate, and propionate levels across three clusters identified by K-Means clustering. For acetate, while the overall Kruskal-Wallis test was not significant (*p* = 0.09, ns), pairwise comparisons suggest a notable difference between cluster 1 and cluster 3 [*p* = 0.017 (*)]. Butyrate levels did not show significant variation across clusters (*p* = 0.22, ns), indicating a more uniform distribution across groups. The Kruskal-Wallis test for propionate [*p* = 0.008 (**)] indicates a statistically significant difference across clusters, with cluster 1 having significantly difference propionate levels compared to cluster 2 [*p* = 0.004 (**)].

For Agglomerative multi clustering analysis presented in Figure 2D, the overall Kruskal-Wallis test was not statistically significant (*p* = 0.07, ns), for acetate, while pairwise comparisons suggest a notable difference between cluster 2 and cluster 3 [*p* = 0.016 (*)]. Butyrate levels remained consistent across all clusters (*p* = 0.29, ns), showing no evidence of differentiation. Propionate [*p* = 0.045 (*)] suggests an overall difference among clusters, with cluster 1 exhibiting significantly higher propionate levels compared to cluster 2 [*p* = 0.029 (*)].

**Figure 3:**
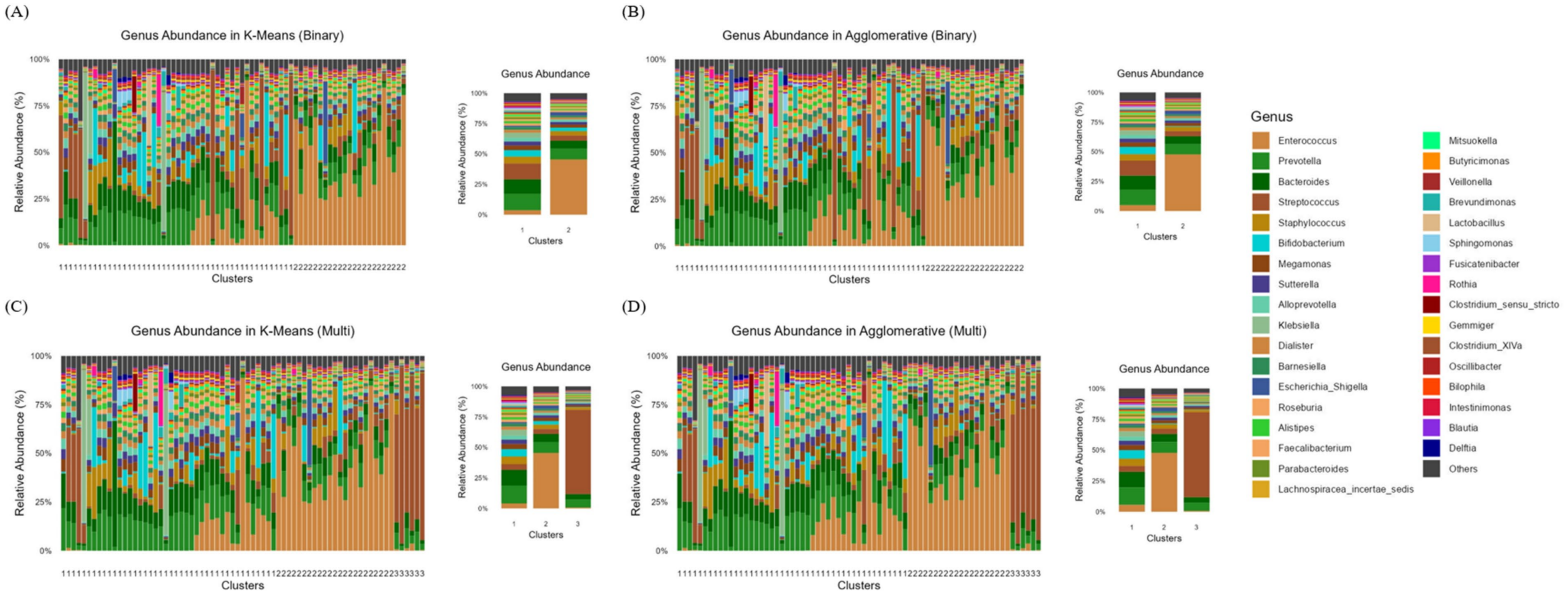
Genus-level relative abundance distributions across clusters identified using K-Means and Agglomerative clustering methods. **(A)** Binary clustering using K-Means, **(B)** Binary clustering using Agglomerative, **(C)** Multi-clustering (three clusters) using K-Means, and **(D)** Multi-clustering using Agglomerative clustering. Each bar represents the relative abundance of different bacterial genera within individual samples, with colors corresponding to the genera listed in the legend.

To further investigate whether specific microbial compositions were associated with variations in SCFAs levels, we analyzed genus-level microbial compositions within each cluster. Figures 3A,B present the genus-level relative abundance distributions obtained using K-Means and Agglomerative clustering under a binary classification scheme. Both clustering methods successfully grouped the microbiome samples into two distinct clusters with noticeable differences in their microbial compositions. The dominant genera in each cluster, suggest that one cluster is enriched in genera such as *Enterococcus, Bacteroides*, and *Prevotella*, while the other cluster shows a more mixed distribution of various genera. The K-Means model (Figure 3A) appears to create a more distinct separation between clusters, with one cluster showing a more uniform genus composition. In contrast, Agglomerative model (Figure 3B) shows a slightly more heterogeneous composition within each cluster, possibly due to its hierarchical nature.

Figures 3C,D display the genus-level relative abundance distributions when clustering was performed with three groups using K-Means and Agglomerative clustering, respectively. The introduction of a third cluster enables finer differentiation of microbial compositions, revealing substructures that were not captured in binary clustering. In K-Means clustering (Figure 3C), cluster 1 is characterized by a high relative abundance of *Enterococcus* and *Bacteroides*, while cluster 2 is dominated by *Prevotella* and *Dialister*. The cluster 3, in contrast, exhibits a more diverse microbiome profile, with *Bifidobacterium*, *Streptococcus*, and *Roseburia* being more prevalent.

Agglomerative clustering (Figure 3D) similarly identifies three clusters, though the transitions between them appear more gradual. The cluster 1 remains enriched in *Enterococcus* and *Bacteroides*, while cluster 2 contains a high proportion of *Prevotella*, *Alloprevotella*, and *Escherichia-Shigella*. The cluster 3, akin to its counterpart in K-Means, shows a more even distribution of multiple genera, with notable contributions from *Streptococcus*, *Lactobacillus*, and *Oscillibacter*.

**Figure 4:**
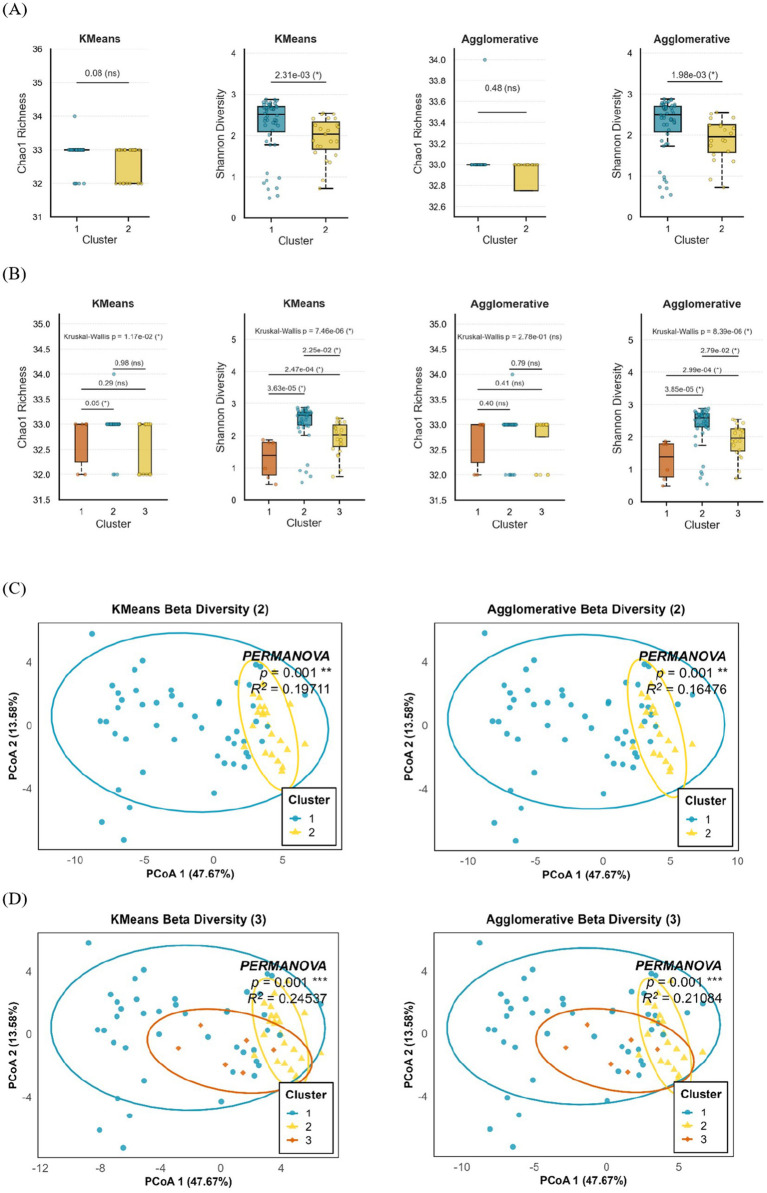
Comparison of neonatal gut microbial diversity and community structure across K-Means and Agglomerative clustering models. **(A,B)** Alpha diversity analysis of neonatal gut microbiota based on **(A)** two-cluster and **(B)** three-cluster models generated by K-Means and Agglomerative clustering. Boxplots display Chao1 richness and Shannon diversity across identified microbial clusters, with Kruskal–Wallis and pairwise Wilcoxon *p*-values shown above each comparison (*p* < 0.05 indicated by *). **(C,D)** Beta diversity analysis using Principal Coordinates Analysis (PCoA) of Bray–Curtis dissimilarity for **(C)** two-cluster and **(D)** three-cluster solutions. Ellipses represent 95% confidence intervals around each cluster centroid. PERMANOVA results are shown in each panel, indicating significant compositional differences between clusters with corresponding *p* values and explained variance (R^2^). Data represent meconium microbiota from 71 infants analyzed at the genus level. Statistical tests were non-parametric (Kruskal–Wallis, Wilcoxon, and PERMANOVA, 999 permutations). Boxplots show median and interquartile range; points denote individual samples.

To evaluate the microbial diversity, we compared Chao1 richness and Shannon diversity across clusters generated by K-Means and Agglomerative clustering models (Figures 4A,B). In the two-cluster models, Shannon diversity was significantly higher in Cluster 2 compared to Cluster 1 (*p* = 2.31 × 10^−3^ for K-Means; *p* = 1.98 × 10^−3^ for Agglomerative), indicating greater evenness and diversity within this group. In contrast, Chao1 richness did not differ significantly between clusters (*p* = 0.08 for K-Means; *p* = 0.48 for Agglomerative), suggesting comparable species richness between the two microbial community structures. In the three-cluster models, similar trends were observed. While Chao1 richness showed modest variation across clusters (*p* = 1.17 × 10^−2^ for K-Means), pairwise comparisons revealed a borderline difference between Clusters 1 and 3 (*p* = 0.05). Shannon diversity, however, exhibited marked differences among clusters (*p* = 7.46 × 10^−6^ for K-Means; *p* = 8.39 × 10^−6^ for Agglomerative), with both methods consistently showing that Cluster 1 had lower microbial diversity than Clusters 2 and 3.

To assess between-sample variation in microbial community composition, beta diversity was evaluated using Bray–Curtis dissimilarity and visualized by PCoA (Figures 4C,D). In the two-cluster models, distinct spatial separation was observed between clusters in both the K-Means and Agglomerative approaches, indicating consistent differentiation in microbial composition. PERMANOVA confirmed these differences to be statistically significant (*p* = 0.001), with R^2^ values of 0.217 for K-Means and 0.200 for Agglomerative clustering, demonstrating that approximately 20–22% of the variance in microbial community structure could be explained by cluster grouping. In the three-cluster models, PCoA plots revealed a clearer segregation of samples, with each cluster occupying distinct compositional space. The PERMANOVA test again demonstrated highly significant differences among clusters (*p* = 0.001) with higher explanatory power (R^2^ = 0.358 for K-Means and R^2^ = 0.343 for Agglomerative), suggesting that three-cluster solutions captured more refined structural variations within the microbial community. These findings collectively indicate that both clustering methods identified distinct microbial community profiles within the neonatal gut. While overall richness remained relatively stable across groups, the observed differences in Shannon diversity and community composition highlight that cluster formation reflects meaningful ecological variation, with certain clusters representing more compositionally diverse and functionally differentiated microbial communities.

**Figure 5:**
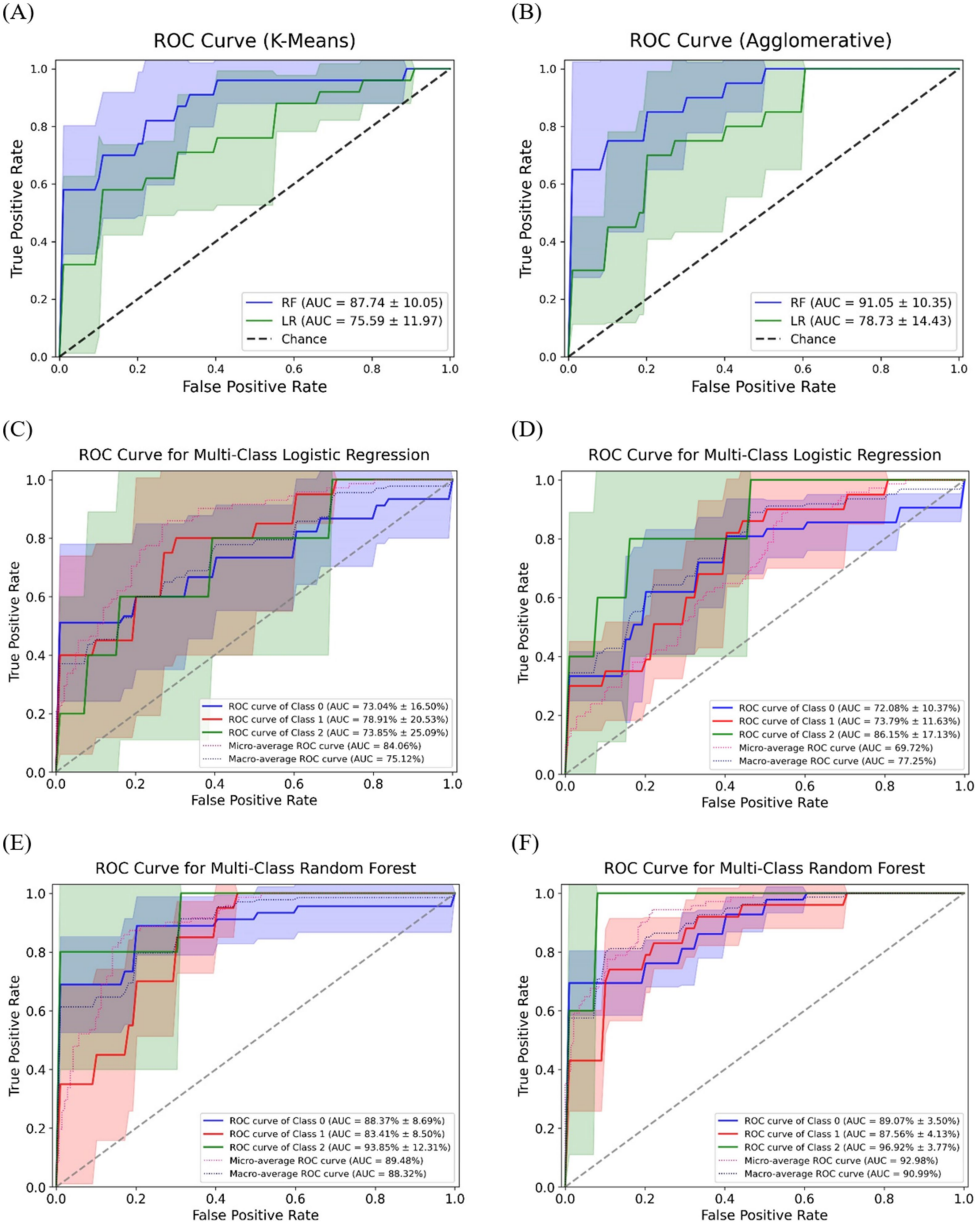
The receiver operating characteristic curves for classification of SCFAs clusters using machine learning models. The ROC curves for binary classification of SCFAs clusters using **(A)** K-Means and **(B)** Agglomerative; Multi-class ROC curves for logistic regression **(C)** K-Means, **(D)** Agglomerative; Multi-class ROC curves for random forest **(E)** K-Means, **(F)** Agglomerative.

The classification accuracy of SCFAs-based clusters was evaluated using LR and RF, as shown in Figure 5. The ROC curves illustrate the predictive performance of models for SCFAs-derived clusters, with K-Means and Agglomerative clustering represented in Figures 5A,B, respectively. The RF consistently demonstrating superior classification performance compared to LR. Specifically, in K-Means clustering, RF achieved an AUC of 87.74 ± 10.05, whereas LR exhibited a lower AUC of 75.59 ± 11.97, indicating that RF more effectively distinguished SCFAs-based clusters. Similarly, in Agglomerative clustering, RF again outperformed LR, with an AUC of 91.05 ± 10.35, compared to 78.73 ± 14.43 for LR.

Figures 5C,D illustrate the multi-class classification performance of LR for SCFAs clusters, providing an evaluation of model capability in distinguishing three distinct groups. When applied to K-Means clustering (Figure 5C), LR achieved a micro-average AUC of 84.06% and a macro-average AUC of 75.12%, indicating moderate discriminative ability. In contrast, its application to Agglomerative clustering (Figure 5D) yielded slightly higher performance, with a micro-average AUC of 86.72% and a macro-average AUC of 77.25%. Overall, LR was able to differentiate SCFA-based clusters, though performance varied by clustering approach, with Agglomerative clustering providing marginally superior accuracy.

In comparison, the RF model (Figures 5E,F) demonstrated superior accuracy and stronger discriminative power across all SCFA-based clusters. For K-Means clustering (Figure 5E), RF achieved a micro-average AUC of 89.84% and a macro-average AUC of 88.32%, surpassing LR. When applied to Agglomerative clustering (Figure 5F), performance improved further, with a micro-average AUC of 92.98% and a macro-average AUC of 90.99%, confirming RF’s greater robustness in distinguishing SCFA-driven microbial community structures.

## Discussion

In this study, we applied unsupervised learning to cluster neonatal gut microbiota based on SCFAs production profiles. Four well-known clustering algorithms were systematically compared using multiple validation metrics (Silhouette Score, Calinski–Harabasz Index, Davies–Bouldin Index, and Prediction Strength). This comprehensive evaluation identified the most effective approach for capturing meaningful microbial subgroups. Given the dataset size, both binary and three-class clustering strategies were tested, with K-Means and Agglomerative consistently outperforming other models. Cluster separation was further validated using t-SNE visualization and genus-level composition analysis to identify key taxa associated with differential SCFA production.

Infants were subsequently assigned to these clusters based on their SCFA profiles to examine the relationship between microbial structure and metabolic output. This analysis revealed how distinct microbial subgroups influence SCFA production and metabolic differentiation. Finally, we evaluated the extent to which these microbiome-driven clusters produced statistically significant differences in SCFAs levels. To evaluate the predictive power of microbial composition in SCFAs metabolism, we applied ML models (LR and RF) using infant demographic and clinical data combined with three SCFAs variables. Overall, the study provides insights into early microbial colonization, metabolic variability, and functional clustering, advancing understanding of neonatal gut development and potential microbiome-based biomarkers.

Clustering performance at the genus level showed that Agglomerative and K-Means consistently outperformed other models across all validation metrics. Centroid-based and hierarchical approaches more effectively captured microbial community structures than density-based or probabilistic methods. Evaluation metrics confirmed the robustness of a three-cluster solution, particularly for Agglomerative clustering, indicating well-separated microbial subgroups. Prediction strength analysis further validated this model, with Agglomerative maintaining slightly higher stability than K-Means at *K* = 3. Beyond three clusters, prediction strength declined sharply, indicating over-segmentation and reduced interpretability. Overall, these results highlight that three clusters represent the most stable and biologically meaningful classification.

The t-SNE visualizations confirmed the validity of our clustering approach, showing clear separations in both two-cluster and three-cluster solutions. Agglomerative clustering better preserved microbial community relationships, while K-Means formed more compact clusters, likely due to its centroid-based partitioning, which may impose artificial boundaries on fluid microbial ecosystems (Lozupone et al., 2012). The emergence of a third cluster suggests a transitional or metabolically distinct microbial community, supporting the idea that microbial composition exists along a functional gradient rather than rigid classifications (Pasolli et al., 2019). This aligns with metagenomic and ecological studies, which propose that microbial populations undergo gradual shifts rather than abrupt separations, influenced by dietary, environmental, and host factors (Gibbons et al., 2017).

The diversity analyses further reinforced the ecological validity of the identified clusters. Although overall species richness, as reflected by the Chao1 index, remained relatively stable among groups, the significant variation in Shannon diversity and Bray–Curtis dissimilarity indicates differences in community evenness and compositional heterogeneity across clusters. These findings indicate that clustering revealed distinct ecological subgroups within the neonatal gut, with some communities showing greater balance and functional diversity. The strong beta diversity separation and higher explanatory power of the three-cluster model suggest that early microbial colonization follows a structured pattern linked to metabolic potential and host–microbiome interactions.

We analyzed SCFAs concentrations (acetate, butyrate, and propionate) across microbial clusters to investigate how these microbiomes extracted structure correlates with metabolic function. In early life, the neonatal gut microbiome rapidly evolves under the influence of factors like delivery mode, maternal microbiota, and feeding patterns(Timmerman et al., 2017), with early microbial colonizers shaping metabolic development by fermenting complex polysaccharides into SCFAs, the primary microbial metabolites in the colon (Timmerman et al., 2017; Macfarlane and Macfarlane, 2012; Markowiak-Kopeć and Śliżewska, 2020). Our binary clustering analysis using K-Means and Agglomerative clustering revealed a distinct stratification of SCFAs levels, with cluster 1 displaying significantly higher concentrations and cluster 2 showing markedly lower levels. The microbial composition of these clusters offers key insights into their metabolic activity, as cluster 1 was enriched with SCFAs-producing bacteria, particularly *Prevotella*, *Bacteroides*, and *Streptococcus*, while cluster 2 was dominated by *Enterococcus* and exhibited a reduced presence of *Prevotella* and *Bacteroides*.

The predominance of *Enterococcus* in Cluster 2 aligns with findings by Al-Balawi & Morsy (Al-Balawi and Morsy, 2020), who identified *Enterococcus faecalis* and *Enterococcus faecium* as early neonatal colonizers, with *E. faecalis* comprising 62.5% of total lactic acid bacteria (LAB). Their study highlights the role of *Enterococcus* in the initial gut microbiota establishment, consistent with our observation that *Enterococcus*-dominant clusters exhibited lower SCFAs production, likely due to its distinct metabolic pathways compared to SCFAs-producing genera. Statistical analysis further confirmed the microbial influence on SCFAs metabolism.

In K-Means clustering, propionate levels differed significantly (*p* = 0.010), with acetate showing a trend toward significance (*p* = 0.06), while butyrate differences were not statistically significant. In contrast, Agglomerative clustering revealed a significant difference in acetate levels (*p* = 0.041), whereas propionate (*p* = 0.05) and butyrate (*p* = 0.22) did not reach significance. The significant acetate variation observed in Agglomerative clustering but not in K-Means suggests that hierarchical clustering may better capture microbial relationships influencing acetate production variability. This aligns with findings by Bäckhed et al. (2015), who reported that early colonizers such as *Bacteroides* were less frequently transmitted from mother to infant, while *Enterococcus faecalis* was more stably transmitted across birth modes. Given that our low-SCFAs cluster was dominated by *Enterococcus*, this may indicate that its metabolic contributions differ from those of *Prevotella* and *Bacteroides*, which were enriched in the high-SCFAs cluster and are well-established SCFAs producers (Martin et al., 2016).

Expanding the analysis to multi-class clustering revealed a third intermediate cluster in both K-Means and Agglomerative clustering, suggesting a potential metabolic transition between the two extremes of SCFAs profiles. While cluster 1 consistently exhibited the highest SCFAs levels and cluster 2 maintained the lowest, cluster 3 emerged as a transitional group with moderate SCFAs concentrations, bridging the metabolic divide between the two polar profiles. Taxonomic analysis revealed distinct microbial compositions across the three clusters. The cluster 1 remained predominantly composed of *Prevotella* and *Bacteroides*, while cluster 2 was largely dominated by *Enterococcus*. In contrast, cluster 3 exhibited a mixed profile, primarily consisting of *Streptococcus* with some *Prevotella* presence. The emergence of this intermediate cluster aligns with the findings of Martin et al. (2016), who highlighted the pivotal role of *Bacteroides* in early gut microbiota development, shaped by perinatal factors such as mode of delivery and diet. This reinforces the dynamic nature of early microbial colonization and its metabolic consequences.

Testing the three-cluster model revealed that differences in acetate and butyrate levels were largely non-significant, with the exception of propionate, which showed statistically significant variation in both K-Means (*p* = 0.008) and Agglomerative clustering (*p* = 0.045). Pairwise comparisons further underscored significant differences: acetate levels in cluster 3 were notably distinct from those in Cluster 2 (*p* = 0.017 in K-Means, *p* = 0.016 in Agglomerative), while propionate levels in cluster 1 were significantly higher than those in cluster 2 (*p* = 0.004 in K-Means, *p* = 0.029 in Agglomerative). The absence of significant differences in butyrate levels suggests that butyrate metabolism may be less susceptible to microbial compositional shifts compared to acetate and propionate. This finding is consistent with Tamana et al. (2021), who reported that a higher relative abundance of *Bacteroides* at one year was linked to improved neurodevelopmental outcomes. Similarly, Carlson et al. (2018) observed that infants enriched with *Bacteroides* at 12 months demonstrated enhanced cognitive development by the age of two. Given that cluster 1 in our study was enriched with *Bacteroides* and *Prevotella*, these results suggest that microbial SCFAs production may have far-reaching implications beyond metabolic health, potentially playing a role in shaping neurodevelopmental trajectories.

These findings highlight the broader significance of microbial clustering, suggesting potential microbiome-targeted interventions. The enrichment of Prevotella and Bacteroides in high-SCFAs clusters aligns with their known role in SCFAs synthesis, while the dominance of *Enterococcus* in low-SCFAs clusters points to alternative metabolic pathways or reduced SCFAs production. This is particularly relevant given Makino et al. (2013), who reported reduced *Bacteroides* transmission in C-section-delivered infants, emphasizing the influence of birth mode on neonatal microbiota. Furthermore, *Bacteroides* depletion has been associated with neurodevelopmental disorders, including autism spectrum disorder (Dan et al., 2020), with lower abundance linked to cognitive and behavioral alterations in early childhood (Carlson et al., 2018; Wang et al., 2017). Additionally, *Bacteroides-Prevotella* ratios have been correlated with infant weight and ponderal index at one month (Obermajer et al., 2017), underscoring the critical role of microbial composition in early metabolic health.

The classification analysis of SCFA-based clusters showed that the RF model consistently outperformed LR in distinguishing cluster structures. The RF achieved superior performance in both K-Means and Agglomerative models, particularly the latter, which yielded the highest ROC scores. This reflects RF model ability to capture nonlinear relationships and complex feature interactions. Multi-class classification further reinforced its advantage, as RF achieved higher micro- and macro-average AUC values than LR, indicating stronger discriminative power across all clusters. Agglomerative clustering also demonstrated slightly higher accuracy than K-Means, suggesting that hierarchical structures better represent biologically meaningful microbial groupings. These findings align with previous studies showing RF model superiority in disease classification tasks. Ripan et al. (2021) reported higher ROC values for RF compared to LR, support vector machine (SVM), and k-nearest neighbors algorithm (KNN) in heart disease prediction. Similarly, Hu et al. (2024) found that KNN outperformed SVM and LR in cardiovascular disease detection. In our study, RF achieved the best overall performance AUCs of 91.05 ± 10.35 for Agglomerative and 87.74 ± 10.05 for K-Means in binary classification, and 92.98% (micro-average) and 90.99% (macro-average) in multi-class classification highlighting RF’s robustness for microbiome-based cluster prediction.

Strengths of this study rely on its comprehensive and methodologically rigorous approach to understanding neonatal gut microbiome dynamics by integrating unsupervised clustering, machine learning, and metabolic profiling. It employs four distinct clustering algorithms (K-Means, Agglomerative, Spectral, and GMM), systematically evaluating their performance using multiple clustering validation metrics to identify the most biologically meaningful microbial subgrouping approach. Unlike traditional microbiome studies that focus primarily on taxonomic composition, this study incorporates SCFAs metabolic profiling, providing a functional perspective on microbial community interactions and bridging the gap between microbial composition and metabolic function. Additionally, supervised ML models (RF and LR) are implemented to classify SCFAs-driven clusters, with hyperparameter tuning and cross-validation ensuring robust and reproducible classification results. The findings offer biologically meaningful and clinically relevant insights, particularly regarding the influence of microbial communities on neonatal metabolism, which has implications for early-life gut development, immune regulation, and microbiome-targeted interventions. Furthermore, by benchmarking its classification performance against existing disease clustering models, such as those used in heart disease and cardiovascular disease classification, this study establishes its novelty and broader significance in pediatric microbiome research. Specifically, it provides new insights into early-life gut microbiota development, highlighting potential microbial biomarkers that could inform pediatric disease risk assessment, early diagnostics, and microbiome-targeted therapeutic interventions. Another key strength of this study is the use of multi-class clustering, which captures gradual metabolic transitions between microbial communities, rather than forcing rigid binary classifications. Additionally, this study enhances biological interpretability by integrating taxonomic and metabolic data, ensuring that the clustering outputs align with known microbial functions. Lastly, it provides a foundation for future longitudinal microbiome research, offering a framework for tracking microbial development and its long-term impact on metabolic health, ultimately paving the way for early disease detection and microbiome-targeted therapeutic strategies in neonates. This finding provides novel evidence that microbial community signatures alone can reveal underlying metabolic and clinical heterogeneity, supporting the potential of microbiome-based unsupervised models to predict functional states such as SCFA production capacity.

Despite its strengths, this study has several limitations. First, the analysis is cross-sectional, providing a static snapshot of microbial composition and SCFAs production, rather than tracking longitudinal changes in microbiome development over time. Second, while clustering effectively identifies microbial subgroups, it does not directly assess functional gene expression or metabolic activity, limiting our ability to confirm whether observed SCFAs variations result from differential microbial metabolism or external environmental factors. Third, although 16S rRNA sequencing provided valuable insights into taxonomic composition, it lacks species-level and functional resolution; future studies integrating shotgun metagenomics and metabolomics are warranted to comprehensively elucidate microbial functions and metabolic pathways. Additionally, sample size constraints may limit the generalizability of findings, requiring validation in larger and more diverse neonatal cohorts. Finally, while random forest demonstrated superior classification performance, alternative deep learning approaches could be explored to further enhance predictive accuracy. Future research should address these limitations by incorporating longitudinal data, functional genomic analyses, and expanded machine learning models to improve the resolution and applicability of microbiome-based SCFAs classification.

## Conclusion

This study provides valuable insights into microbiome-based decision-making in neonatal health by demonstrating the potential of machine learning-driven clustering and classification models for stratifying microbial subgroups based on SCFA metabolic profiles. The findings emphasize the broader applicability of microbiome informatics in clinical and public health settings, offering a foundation for early microbiome-targeted interventions. Integrating SCFA-based microbiome classification with biomedical informatics systems could enhance predictive healthcare models, allowing clinicians to identify neonates at higher risk for gastrointestinal and metabolic disorders, optimize personalized nutritional and probiotic strategies, and incorporate microbiome screening into routine neonatal care. From a research perspective, these findings pave the way for longitudinal studies tracking microbial shifts over time, which could refine biomarker discovery for disease risk assessment. Additionally, advanced machine learning models hold promise for improving the predictive accuracy of microbial classification, enabling more precise and automated decision-making in neonatal and pediatric healthcare. Ultimately, this study underscores the clinical and translational potential of microbiome informatics in shaping evidence-based interventions, precision medicine strategies, and microbiome-driven health monitoring systems, fostering a proactive approach to neonatal metabolic and immune health management.

## Data Availability

The datasets presented in this study can be found in online repositories. The names of the repository/repositories and accession number(s) can be found at: https://www.ncbi.nlm.nih.gov/, PRJNA1123597.

## References

[ref1] Abo Kouadio Jerome M. Fatogoma Etienne S. Ngolo David C. Thomas M. Fréderic P. Véronique C. . (2023). Faecal short-chain fatty acid and early introduction of foods in the first 200 days of infant’s life in the district of Abidjan (Ivory Coast). Am. J. Food Nutr. 11, 7–15. doi: 10.12691/ajfn-11-1-2

[ref2] Ahn J. Hayes R. B. (2021). Environmental influences on the human microbiome and implications for noncommunicable disease. Annu. Rev. Public Health 42, 277–292. doi: 10.1146/annurev-publhealth-012420-105020, PMID: 33798404 PMC8641399

[ref3] Al-Balawi M. Morsy F. M. (2020). *Enterococcus faecalis* is a better competitor than other lactic acid bacteria in the initial colonization of colon of healthy newborn babies at first week of their life. Front. Microbiol. 11:2017. doi: 10.3389/fmicb.2020.02017/full 33133027 PMC7550472

[ref4] Arlot S. Celisse A. (2010). A survey of cross-validation procedures for model selection. Stat. Surv. 4, 40–79. doi: 10.1214/09-SS054

[ref5] Bäckhed F. Roswall J. Peng Y. Feng Q. Jia H. Kovatcheva-Datchary P. . (2015). Dynamics and stabilization of the human gut microbiome during the first year of life. Cell Host Microbe 17, 690–703. doi: 10.1016/j.chom.2015.04.004, PMID: 25974306

[ref6] Barman M. Gio-Batta M. Andrieux L. Stråvik M. Saalman R. Fristedt R. . (2024). Short-chain fatty acids (SCFA) in infants’ plasma and corresponding mother’s milk and plasma in relation to subsequent sensitisation and atopic disease. EBioMedicine 101:104999. doi: 10.1016/j.ebiom.2024.104999, PMID: 38340558 PMC10869761

[ref7] Bridgman S. L. Azad M. B. Field C. J. Haqq A. M. Becker A. B. Mandhane P. J. . (2017). Fecal short-chain fatty acid variations by breastfeeding status in infants at 4 months: differences in relative versus absolute concentrations. Front. Nutr. 4:11. doi: 10.3389/fnut.2017.00011 28443284 PMC5385454

[ref8] Byrne C. S. Chambers E. S. Morrison D. J. Frost G. (2015). The role of short chain fatty acids in appetite regulation and energy homeostasis. Int. J. Obes. 39, 1331–1338. doi: 10.1038/ijo.2015.84, PMID: 25971927 PMC4564526

[ref9] Cai Y. Gu H. Kenney T. (2017). Learning microbial community structures with supervised and unsupervised non-negative matrix factorization. Microbiome. 5:110. doi: 10.1186/s40168-017-0323-1, PMID: 28859695 PMC5579944

[ref10] Cai Y. Sun Y. (2011). ESPRIT-tree: hierarchical clustering analysis of millions of 16S rRNA pyrosequences in quasilinear computational time. Nucleic Acids Res. 39:e95. doi: 10.1093/nar/gkr349, PMID: 21596775 PMC3152367

[ref11] Carlson A. L. Xia K. Azcarate-Peril M. A. Goldman B. D. Ahn M. Styner M. A. . (2018). Infant gut microbiome associated with cognitive development. Biol. Psychiatry 83, 148–159. doi: 10.1016/j.biopsych.2017.06.021, PMID: 28793975 PMC5724966

[ref12] Chun J. Toldi G. (2022). The impact of short-chain fatty acids on neonatal regulatory T cells. Nutrients 14:3670. doi: 10.3390/nu14183670, PMID: 36145046 PMC9503436

[ref13] Cifuentes M. P. Chapman J. A. Stewart C. J. (2024). Gut microbiome derived short chain fatty acids: promising strategies in necrotising enterocolitis. Curr Res Microb Sci. 6:100219. doi: 10.1016/j.crmicr.2024.100219, PMID: 38303965 PMC10831176

[ref14] Collado M. C. Rautava S. Aakko J. Isolauri E. Salminen S. (2016). Human gut colonisation may be initiated in utero by distinct microbial communities in the placenta and amniotic fluid. Sci. Rep. 6, 6:23129. doi: 10.1038/srep23129, PMID: 27001291 PMC4802384

[ref15] Cox D. R. (1958). The regression analysis of binary sequences. J. R. Stat. Soc. Ser. B 20, 215–242.

[ref16] Dai X. Yuan T. Zhang X. Zhou Q. Bi H. Yu R. . (2020). Short-chain fatty acid (SCFA) and medium-chain fatty acid (MCFA) concentrations in human milk consumed by infants born at different gestational ages and the variations in concentration during lactation stages. Food Funct. 11, 1869–1880. doi: 10.1039/C9FO02595B, PMID: 32068229

[ref17] Dan Z. Mao X. Liu Q. Guo M. Zhuang Y. Liu Z. . (2020). Altered gut microbial profile is associated with abnormal metabolism activity of autism Spectrum disorder. Gut Microbes 11, 1246–1267. doi: 10.1080/19490976.2020.1747329, PMID: 32312186 PMC7524265

[ref18] den Besten G. van Eunen K. Groen A. K. Venema K. Reijngoud D.-J. Bakker B. M. (2013). The role of short-chain fatty acids in the interplay between diet, gut microbiota, and host energy metabolism. J. Lipid Res. 54, 2325–2340. doi: 10.1194/jlr.R036012, PMID: 23821742 PMC3735932

[ref19] Dominguez-Bello M. G. Costello E. K. Contreras M. Magris M. Hidalgo G. Fierer N. . (2010). Delivery mode shapes the acquisition and structure of the initial microbiota across multiple body habitats in newborns. Proc. Natl. Acad. Sci. 107, 11971–11975. doi: 10.1073/pnas.1002601107, PMID: 20566857 PMC2900693

[ref20] Favari C. Rinaldi de Alvarenga J. F. Sánchez-Martínez L. Tosi N. Mignogna C. Cremonini E. . (2024). Factors driving the inter-individual variability in the metabolism and bioavailability of (poly)phenolic metabolites: A systematic review of human studies. Redox Biol. 71:103095. doi: 10.1016/j.redox.2024.103095, PMID: 38428187 PMC10912651

[ref21] Gacesa R. Kurilshikov A. Vich Vila A. Sinha T. Klaassen M. A. Y. Bolte L. A. . (2022). Environmental factors shaping the gut microbiome in a Dutch population. Nature 604, 732–739. doi: 10.1038/s41586-022-04567-7 35418674

[ref22] Gao Y. Davis B. Zhu W. Zheng N. Meng D. Walker W. A. (2021). Short-chain fatty acid butyrate, a breast milk metabolite, enhances immature intestinal barrier function genes in response to inflammation *in vitro* and *in vivo*. Am. J. Physiol. Liver Physiol. 320, G521–G530. doi: 10.1152/ajpgi.00279.2020 PMC8238162 33085904

[ref24] Gibbons S. M. Kearney S. M. Smillie C. S. Alm E. J. (2017). Two dynamic regimes in the human gut microbiome. PLoS Comput. Biol. 13:e1005364. doi: 10.1371/journal.pcbi.1005364 28222117 PMC5340412

[ref25] Gio-Batta M. Spetz K. Barman M. Bråbäck L. Norin E. Björkstén B. . (2022). Low concentration of Fecal Valeric acid at 1 year of age is linked with eczema and food allergy at 13 years of age: findings from a Swedish birth cohort. Int. Arch. Allergy Immunol. 183, 398–408. doi: 10.1159/000520149, PMID: 34839288 PMC9153367

[ref26] Grier A. Qiu X. Bandyopadhyay S. Holden-Wiltse J. Kessler H. A. Gill A. L. . (2017). Impact of prematurity and nutrition on the developing gut microbiome and preterm infant growth. Microbiome. 5:158. doi: 10.1186/s40168-017-0377-0, PMID: 29228972 PMC5725645

[ref27] Guo W. Zhang Z. Li L. Liang X. Wu Y. Wang X. . (2022). Gut microbiota induces DNA methylation via SCFAs predisposing obesity-prone individuals to diabetes. Pharmacol. Res. 182:106355. doi: 10.1016/j.phrs.2022.106355 35842183

[ref28] Guzzardi M. A. Ait Ali L. D’Aurizio R. Rizzo F. Saggese P. Sanguinetti E. . (2019). Fetal cardiac growth is associated with in utero gut colonization. Nutr. Metab. Cardiovasc. Dis. 29, 170–176. doi: 10.1016/j.numecd.2018.10.005 30579777

[ref29] Harris C. R. Millman K. J. van der Walt S. J. Gommers R. Virtanen P. Cournapeau D. . (2020). Array programming with NumPy. Nature 585, 357–362. doi: 10.1038/s41586-020-2649-2, PMID: 32939066 PMC7759461

[ref30] Heath A.-L. M. Haszard J. J. Galland B. C. Lawley B. Rehrer N. J. Drummond L. N. . (2020). Association between the faecal short-chain fatty acid propionate and infant sleep. Eur. J. Clin. Nutr. 74, 1362–1365. doi: 10.1038/s41430-019-0556-0, PMID: 31969698

[ref31] Hernández-Martínez C. Canals J. Voltas N. Martín-Luján F. Arija V. (2022). Circulating levels of short-chain fatty acids during pregnancy and infant neurodevelopment. Nutrients 14:3946. doi: 10.3390/nu14193946, PMID: 36235606 PMC9573109

[ref32] Ho T. K. (1995). “Random decision forests” in Proceedings of 3rd international conference on document analysis and recognition (Computer Society Press) 1, 278–282. doi: 10.1109/ICDAR.1995.598994

[ref33] Hsu C.-Y. Khachatryan L. G. Younis N. K. Mustafa M. A. Ahmad N. Athab Z. H. . (2024). Microbiota-derived short chain fatty acids in pediatric health and diseases: from gut development to neuroprotection. Front. Microbiol. 15:1456793. doi: 10.3389/fmicb.2024.1456793 39439941 PMC11493746

[ref34] Hu Y. Yan H. Liu M. Gao J. Xie L. Zhang C. . (2024). Detecting cardiovascular diseases using unsupervised machine learning clustering based on electronic medical records. BMC Med. Res. Methodol. 24:309. doi: 10.1186/s12874-024-02422-z, PMID: 39702064 PMC11658374

[ref35] Jiménez E. Fernández L. Marín M. L. Martín R. Odriozola J. M. Nueno-Palop C. . (2005). Isolation of commensal bacteria from umbilical cord blood of healthy neonates born by Cesarean section. Curr. Microbiol. 51, 270–274. doi: 10.1007/s00284-005-0020-3, PMID: 16187156

[ref36] Koh A. De Vadder F. Kovatcheva-Datchary P. Bäckhed F. (2016). From dietary Fiber to host physiology: short-chain fatty acids as key bacterial metabolites. Cell 165, 1332–1345. doi: 10.1016/j.cell.2016.05.041, PMID: 27259147

[ref37] Kopczyńska J. Kowalczyk M. (2024). The potential of short-chain fatty acid epigenetic regulation in chronic low-grade inflammation and obesity. Front. Immunol. 15:1380476. doi: 10.3389/fimmu.2024.1380476 38605957 PMC11008232

[ref38] Kwon Y. Cho K. H. Ma S. Ko H. Hong G.-H. Lee S.-Y. . (2024). Supplementation of heat-treated Lactiplantibacillus plantarum nF1 changes the production of short-chain fatty acids in healthy infants. J Nutr Metab. 2024, 1–8. doi: 10.1155/2024/5558566, PMID: 38623309 PMC11018375

[ref39] Lahti L Shetty S. (2017). Microbiome R package. doi: 10.18129/B9.bioc.microbiome

[ref40] Liu P. Wang Y. Yang G. Zhang Q. Meng L. Xin Y. . (2021). The role of short-chain fatty acids in intestinal barrier function, inflammation, oxidative stress, and colonic carcinogenesis. Pharmacol. Res. 165:105420. doi: 10.1016/j.phrs.2021.105420, PMID: 33434620

[ref41] Łoniewska B. Fraszczyk-Tousty M. Tousty P. Skonieczna-Żydecka K. Maciejewska-Markiewicz D. Łoniewski I. (2023). Analysis of Fecal short-chain fatty acids (SCFAs) in healthy children during the first two years of life: an observational prospective cohort study. Nutrients 15:367. doi: 10.3390/nu15020367, PMID: 36678236 PMC9864378

[ref42] Lozupone C. A. Stombaugh J. I. Gordon J. I. Jansson J. K. Knight R. (2012). Diversity, stability and resilience of the human gut microbiota. Nature 489, 220–230. doi: 10.1038/nature11550, PMID: 22972295 PMC3577372

[ref43] Luo P. Lednovich K. Xu K. Nnyamah C. Layden B. T. Xu P. (2022). Central and peripheral regulations mediated by short-chain fatty acids on energy homeostasis. Transl. Res. 248, 128–150. doi: 10.1016/j.trsl.2022.06.003, PMID: 35688319 PMC12553404

[ref44] Luo M. Wong S. Chen X. Zhou Z. Du H. Han Y. . (2025). Unveiling interindividual variability in gut microbiota-mediated curcumin metabolism. Food Biosci. 65:105941. doi: 10.1016/j.fbio.2025.105941

[ref45] Macfarlane G. T. Macfarlane S. (2012). Bacteria, colonic fermentation, and gastrointestinal health. J. AOAC Int. 95, 50–60. doi: 10.5740/jaoacint.SGE_Macfarlane, PMID: 22468341

[ref46] Maher S. E. O’Brien E. C. Moore R. L. Byrne D. F. Geraghty A. A. Saldova R. . (2023). The association between the maternal diet and the maternal and infant gut microbiome: a systematic review. Br. J. Nutr. 129, 1491–1499. doi: 10.1017/S0007114520000847, PMID: 32129734

[ref47] Makino H. Kushiro A. Ishikawa E. Kubota H. Gawad A. Sakai T. . (2013). Mother-to-infant transmission of intestinal Bifidobacterial strains has an impact on the early development of vaginally delivered infant’s microbiota. PLoS One 8:e78331. doi: 10.1371/journal.pone.0078331, PMID: 24244304 PMC3828338

[ref48] Markowiak-Kopeć P. Śliżewska K. (2020). The effect of probiotics on the production of short-chain fatty acids by human intestinal microbiome. Nutrients 12:1107. doi: 10.3390/nu12041107, PMID: 32316181 PMC7230973

[ref49] Martin R. Makino H. Cetinyurek Yavuz A. Ben-Amor K. Roelofs M. Ishikawa E. . (2016). Including mode of delivery and type of feeding, siblings and gender, shape the developing gut microbiota. PLoS One 11:e0158498. doi: 10.1371/journal.pone.0158498, PMID: 27362264 PMC4928817

[ref50] McKinney W. O. (2010). Data structures for statistical computing in python. Proc 9th Python Sci Conf 445, 51–56.

[ref51] McMurdie P. J. Holmes S. (2013). Phyloseq: an R package for reproducible interactive analysis and graphics of microbiome census data. PLoS One 8:e61217. doi: 10.1371/journal.pone.0061217 23630581 PMC3632530

[ref52] Milani C. Duranti S. Bottacini F. Casey E. Turroni F. Mahony J. . (2017). The first microbial colonizers of the human gut: composition, activities, and health implications of the infant gut microbiota. Microbiol. Mol. Biol. Rev. 81. doi: 10.1128/MMBR.00036-17, PMID: 29118049 PMC5706746

[ref53] Mshvildadze M. Neu J. Shuster J. Theriaque D. Li N. Mai V. (2010). Intestinal microbial ecology in premature infants assessed with non–culture-based techniques. J. Pediatr. 156, 20–25. doi: 10.1016/j.jpeds.2009.06.063, PMID: 19783002 PMC3628625

[ref54] Mueller Andreas MK. (n.d.). The:mod:`sklearn.Utils.Class_weight` module includes utilities for handling weights based on class labels. Available online at: https://scikit-learn.org/stable/modules/generated/sklearn.utils.class_weight.compute_class_weight.html#sklearn.utils.class_weight.compute_class_weight

[ref55] Mueller N. Differding M. Østbye T. Hoyo C. Benjamin-Neelon S. (2021). Association of birth mode of delivery with infant faecal microbiota, potential pathobionts, and short chain fatty acids: a longitudinal study over the first year of life. BJOG 128, 1293–1303. doi: 10.1111/1471-0528.16633 33338292 PMC8211907

[ref56] Nshanian M. Gruber J. J. Geller B. S. Chleilat F. Lancaster S. M. White S. M. . (2025). Short-chain fatty acid metabolites propionate and butyrate are unique epigenetic regulatory elements linking diet, metabolism and gene expression. Nat. Metab. 7, 196–211. doi: 10.1038/s42255-024-01191-9, PMID: 39789354 PMC11774759

[ref57] Obermajer T. Grabnar I. Benedik E. Tušar T. Robič Pikel T. Fidler Mis N. . (2017). Microbes in infant gut development: placing abundance within environmental, clinical and growth parameters. Sci. Rep. 7:11230. doi: 10.1038/s41598-017-10244-x, PMID: 28894126 PMC5593852

[ref58] Oksanen J Simpson GL Blanchet FG Kindt R Legendre P Minchin PR . (2001). Vegan: community ecology package. CRAN: contributed packages. Available online at: https://cran.r-project.org/package=vegan

[ref59] Parada Venegas D. De la Fuente M. K. Landskron G. González M. J. Quera R. Dijkstra G. . (2019). Short chain fatty acids (SCFAs)-mediated gut epithelial and immune regulation and its relevance for inflammatory bowel diseases. Front. Immunol. 10:10. doi: 10.3389/fimmu.2019.00277/full 30915065 PMC6421268

[ref60] Pasolli E. Asnicar F. Manara S. Zolfo M. Karcher N. Armanini F. . (2019). Extensive unexplored human microbiome diversity revealed by over 150,000 genomes from metagenomes spanning age, geography, and lifestyle. Cell 176, 649–662.e20. doi: 10.1016/j.cell.2019.01.001, PMID: 30661755 PMC6349461

[ref61] Pedregosa F. Varoquaux G. Gramfort A. Michel V. Thirion B. Grisel O. . (2011). Scikit-learn: machine learning in python. J. Mach. Learn. Res.

[ref62] Portincasa P. Bonfrate L. Vacca M. De Angelis M. Farella I. Lanza E. . (2022). Gut microbiota and short chain fatty acids: implications in glucose homeostasis. Int. J. Mol. Sci. 23:1105. doi: 10.3390/ijms23031105, PMID: 35163038 PMC8835596

[ref63] R Core Team. (2020). R: A language and environment for statistical computing. R foundation for statistical computing. Vienna, Austria. Available online at: https://www.r-project.org/

[ref64] Reitmeier S. Kiessling S. Clavel T. List M. Almeida E. L. Ghosh T. S. . (2020). Arrhythmic gut microbiome signatures predict risk of type 2 diabetes. Cell Host Microbe 28, 258–272.e6. doi: 10.1016/j.chom.2020.06.004, PMID: 32619440

[ref65] Ripan R. C. Sarker I. H. Hasan Furhad M. Musfique Anwar M. Hoque M. M. (2021). “An effective heart disease prediction model based on machine learning techniques BT - hybrid intelligent systems” In: eds. Abraham A. Hanne T. Castillo O. Gandhi N. Nogueira Rios T. Hong T. P. (Cham: Springer International Publishing), 280–288.

[ref23] Sharma G Colantuoni C. Goff L. A Fertig E. J. Stein-O’Brien G. (2020). GS-O. ProjectR: an R/bioconductor package for transfer learning via PCA, NMF, correlation and clustering. Bioinformatics. 36, 3592–3593. doi: 10.1093/bioinformatics/btaa183 32167521 PMC7267840

[ref66] Tamana S. K. Tun H. M. Konya T. Chari R. S. Field C. J. Guttman D. S. . (2021). Bacteroides-dominant gut microbiome of late infancy is associated with enhanced neurodevelopment. Gut Microbes 13, 1–17, PMID: 34132157 10.1080/19490976.2021.1930875 PMC8210878

[ref67] Tamburini S. Shen N. Wu H. C. Clemente J. C. (2016). The microbiome in early life: implications for health outcomes. Nat. Med. 22, 713–722. doi: 10.1038/nm.4142, PMID: 27387886

[ref68] Tan P. N. (2009). “Receiver operating characteristic” in Encyclopedia of database systems (Boston, MA: Springer US), 2349–2352.

[ref69] Timmerman H. M. Rutten N. B. M. M. Boekhorst J. Saulnier D. M. Kortman G. A. M. Contractor N. . (2017). Intestinal colonisation patterns in breastfed and formula-fed infants during the first 12 weeks of life reveal sequential microbiota signatures. Sci. Rep. 7:8327. doi: 10.1038/s41598-017-08268-4, PMID: 28827640 PMC5567133

[ref70] Turnbaugh P. J. Hamady M. Yatsunenko T. Cantarel B. L. Duncan A. Ley R. E. . (2009). A core gut microbiome in obese and lean twins. Nature 457, 480–484. doi: 10.1038/nature07540, PMID: 19043404 PMC2677729

[ref71] Van Rossum G. Drake F. L. (2009). Python 3 reference manual. Scotts Valley, CA: CreateSpace.

[ref72] Wang J. Fu K. Chen L. Duan X. Guo X. Chen H. . (2017). Increased gray matter volume and resting-state functional connectivity in somatosensory cortex and their relationship with autistic symptoms in young boys with autism spectrum disorder. Front. Physiol. 8:588:10.3389/fphys.2017.00588 28861001 10.3389/fphys.2017.00588 PMC5559537

[ref73] Wang X. Li J. Li N. Guan K. Yin D. Zhang H. . (2021). Evolution of intestinal gases and fecal short-chain fatty acids produced *in vitro* by preterm infant gut microbiota during the first 4 weeks of life. Front. Pediatr. 9:726193. doi: 10.3389/fped.2021.726193 34646797 PMC8504453

[ref74] Wickham H. (2016). “Data analysis BT - ggplot2: elegant graphics for data analysis” In ed. Wickham H. (Cham: Springer International Publishing), 189–201.

[ref75] Wickham H. Averick M. Bryan J. Chang W. McGowan L. D. A. François R. . (2019). Welcome to the Tidyverse. J. Open Source Softw. 4:1686. doi: 10.21105/joss.01686

[ref76] Wu H. Tremaroli V. Schmidt C. Lundqvist A. Olsson L. M. Krämer M. . (2020). The gut microbiota in prediabetes and diabetes: A population-based cross-sectional study. Cell Metab. 32, 379–390.e3. doi: 10.1016/j.cmet.2020.06.011, PMID: 32652044

