# Supplementary material for: Neonatal gut microbiota stratification and identification of SCFA-associated microbial subgroups using unsupervised clustering and machine learning classification
Source: Front Microbiol. 2025 Dec 4;16:1668451. doi: 10.3389/fmicb.2025.1668451 (PMC12712340; doi:10.3389/fmicb.2025.1668451)
Supplement: Supplementary file 1 [file Table_1.docx]

Neonatal Gut Microbiota Stratification and Identification of SCFA-Associated Microbial Subgroups Using Unsupervised Clustering and Machine Learning Classification

Payam Hosseinzadeh Kasani^1^, Cheol-Heui Yun^2,3,4^, Kee Hyun Cho^1*^, Su Jin Jeong^5*^

^1^Department of Pediatrics, Kangwon National University Hospital, Kangwon National University School of Medicine, Chuncheon, Republic of Korea

^2^Department of Agricultural Biotechnology, and Research Institute of Agriculture and Life Sciences, Seoul National University, Seoul, Republic of Korea

^3^Center for Food and Bioconvergence, and Interdisciplinary Programs in Agricultural Genomics, Seoul National University, Seoul, Republic of Korea

^4^Institutes of Green Bio Science and Technology, Seoul National University, Pyeongchang, Republic of Korea

^5^Department of Pediatrics, CHA Bundang Medical Center, CHA University School of Medicine, Seongnam, Republic of Korea

**Correspondence:**

Kee Hyun Cho; [neo21@kangwon.ac.kr](mailto:neo21@kangwon.ac.kr)

Su Jin Jeong; [jinped@cha.ac.kr](mailto:jinped@cha.ac.kr)

# Supplementary Material

## Supplementary Table

Supplementary Table 1. Maternal and Neonatal Characteristics by Clustering Method

|  | **K-Means** | |  | **Agglomerative** | |  |
| --- | --- | --- | --- | --- | --- | --- |
| **Feature** | Cluster 1 (n=48) | Cluster 2 (n=23) | *p* | Cluster 1 (n=51) | Cluster 2 (n=20) | *p* |
| Sex (Male) | 43.8% | 30.4% |  | 43.1% | 30.0% |  |
| Delivery mode (C-Section) | 93.8% | 100.0% |  | 94.1% | 100.0% |  |
| Twin (Yes) | 4.2% | 13% |  | 3.9% | 15% |  |
| GDM (Yes) | 12.5% | 4.3% |  | 11.8% | 5.0% |  |
| PIH (Yes) | 6.2% | 0.0% |  | 5.9% | 0.0% |  |
| Gestational age | 38.6 ± 0.8 | 38.3 ± 1.0 |  | 38.3 ± 0.7 | 38.3 ± 1.0 |  |
| Birth Weight | 3182.0 ± 258 | 3153.0 ± 384 |  | 3217.9 ± 295 | 3057.0 ± 296 | ***** |
| maternal age | 34.3 ± 4.3 | 33.8 ± 4.8 |  | 34.2 ± 4.3 | 34.0 ± 4.9 |  |
| maternal BMI | 28.4 ± 4.2 | 27.2 ± 3.3 |  | 28.4 ± 4.3 | 27.0 ± 2.7 |  |
| Acetate | 1190.6 ± 1617 | 280.6 ± 247 | 0.057 | 1147.6 ± 1580 | 253.6 ± 206 | ***** |
| Propionate | 200.9 ± 413 | 8.9 ± 2.1 | ***** | 189.4 ± 403.1 | 9.3 ± 0.9 | ***** |
| Butyrate | 41.1 ± 167 | 9.4 ± 5.7 |  | 39.2 ± 162.1 | 9.5 ± 5.7 |  |

Continuous variables are presented as mean ± SD; categorical variables as percentages within each cluster.

Bold p-values indicate statistical significance (p < 0.05).

P-values were calculated using χ² tests for categorical variables and Mann–Whitney U or Kruskal–Wallis tests for continuous variables. No multiple testing correction was applied.

Supplementary Table 2. Maternal and Neonatal Characteristics by Multi-Class Clustering Method

|  | **K-Means** | | |  | **Agglomerative** | | |  |
| --- | --- | --- | --- | --- | --- | --- | --- | --- |
| **Feature** | 1 (n=42) | 2 (n=23) | 3 (n=6) | *p* | 1 (n=45) | 2 (n=20) | 3 (n=6) | *p* |
| Sex (Male) | 42.9 | 30.4% | 50.0% |  | 42.2% | 30.0% | 50.0% |  |
| Delivery mode (C- section) | 92.9 | 100.0% | 100.0% |  | 93.3% | 100.0% | 100.0% |  |
| Twin (Yes) | 4.8 | 13.0% | 0.0% |  | 4.4% | 15% | 0.0% |  |
| GDM (Yes) | 11.9 | 4.3% | 16.7% |  | 11.1% | 5.0% | 16.7% |  |
| PIH (Yes) | 4.8% | 0.0% | 16.7% |  | 4.4% | 0.0% | 16.7% |  |
| Gestational age | 38.6 ± 0.7 | 38.3 ± 1.0 | 38.6 ± 1.0 |  | 38.6 ± 0.7 | 38.3 ± 1.0 | 38.6 ± 1.0 |  |
| Birth Weight | 3159.6 ± 262.6 | 3153.0 ± 384.8 | 3338.3 ± 174.5 |  | 3201.9 ± 306 | 3057.0 ± 296.8 | 3338.3 ± 174 | ***** |
| maternal age | 34.0 ± 4.5 | 33.8 ± 4.8 | 36.2 ± 2.8 |  | 33.9 ± 4.4 | 34.0 ± 4.9 | 36.2 ± 2.8 |  |
| Maternal BMI at delivery | 28.5 ± 4.4 | 27.2 ± 3.3 | 27.7 ± 2.3 |  | 28.5 ± 4.5 | 27.0 ± 2.7 | 27.7 ± 2.3 |  |
| Acetate | 1203.7 ± 1708.7 | 280.6 ± 247.3 | 1098.3 ± 806.2 |  | 1154.2 ± 1663 | 253.6 ± 206.1 | 1098.3 ± 806 |  |
| Propionate | 228.6 ± 435.0 | 8.9 ± 2.1 | 6.3 ± 4.9 | ***** | 213.8 ± 423 | 9.3 ± 0.9 | 6.3 ± 4.9 | ***** |
| Butyrate | 45.5 ± 178.3 | 9.4 ± 5.7 | 10.2 ± 8.4 |  | 43.0 ± 172.4 | 9.5 ± 5.7 | 10.2 ± 8.4 |  |

Continuous variables are presented as mean ± SD; categorical variables as percentages. Bold p-values indicate statistical significance (p < 0.05).

P-values were calculated using χ² tests for categorical variables and Kruskal–Wallis tests for continuous variables. No multiple testing correction was applied.
